# DNA methylation and hydroxymethylation profiles reveal possible role of highly methylated TLR signaling on *Fasciola gigantica* excretory/secretory products (FgESPs) modulation of buffalo dendritic cells

**DOI:** 10.1186/s13071-019-3615-4

**Published:** 2019-07-23

**Authors:** Xue-Fang Mei, Wei Shi, Yao-Yao Zhang, Bin Zhu, Yu-Rui Wang, Lin-Jing Hou, Wen-Ping Zhao, Jian Li, Dong-Ying Wang, Hong-Lin Luo, Wei-Yi Huang

**Affiliations:** 1 School of Animal Science and Technology, Guangxi University, Nanning, People’s Republic of China; 2 School of Preclinical Medicine, Guangxi Medical University, Nanning, People’s Republic of China; 3 State Key Laboratory of Genetic Engineering, Ministry of Education Key Laboratory of Contemporary Anthropology, Collaborative Innovation Center for Genetics and Development, School of Life Sciences, Fudan University, Shanghai, People’s Republic of China; 4 Guangxi Key Laboratory for Aquatic Genetic Breeding and Healthy Aquaculture, Guangxi Institute of Fishery Sciences, Nanning, People’s Republic of China

**Keywords:** *Fasciola gigantica*, Excretory/secretory products, Dendritic cells, (hydroxy)methylation, (h)MeDIP-Seq

## Abstract

**Background:**

Excretory/secretory products (ESPs) released by parasites influence the development and functions of host dendritic cells (DCs). However, little is known about changes of DNA (hydroxy)methylation on DC development during *Fasciola gigantica* infection. The present study aimed to investigate whether *F. gigantica* ESPs (FgESPs) affects the development and functions of buffalo DCs through altering the DNA (hydroxy)methylation of DCs.

**Methods:**

Buffalo DCs were prepared from peripheral blood mononuclear cells (PBMCs) and characterized using scanning and transmission electron microscopy (SEM/TEM) and quantitative reverse transcriptional PCR (qRT-RCR). DCs were treated with 200 μg/ml of FgESPs *in vitro*, following DNA extraction. The DNA methylome and hydroxymethylome were profiled based on (hydroxy)methylated DNA immunoprecipitation sequencing [(h)MeDIP-Seq] and bioinformatics analyses. qRT-RCR was also performed to assess the gene transcription levels of interest.

**Results:**

FgESPs markedly suppressed DC maturation evidenced by morphological changes and downregulated gene expression of CD1a and MHC II. Totals of 5432 and 360 genes with significant changes in the 5-methylcytosine (5-mC) and the 5-hydroxymethylcytosine (5-hmC) levels, respectively, were identified in buffalo DCs in response to FgESPs challenge. Gene Ontology (GO) and Kyoto Encyclopedia of Genes and Genomes (KEGG) analysis revealed that these differentially expressed genes were highly enriched in pathways associated with immune response. Some cancer-related pathways were also indicated. There were 111 genes demonstrating changes in both 5-mC and 5-hmC levels, 12 of which were interconnected and enriched in 12 pathways. The transcription of hypermethylated genes TLR2, TLR4 and IL-12B were downregulated or in a decreasing trend, while the mRNA level of high-hydroxymethylated TNF gene was upregulated in buffalo DCs post-exposure to FgESPs *in vitro*.

**Conclusions:**

To our knowledge, the present study provides for the first time a unique genome-wide profile of DNA (hydroxy)methylation for DCs that interact with FgESPs, and suggests a possible mechanism of FgESPs in suppressing DC maturation and functions that are involved in TLR signaling.

**Electronic supplementary material:**

The online version of this article (10.1186/s13071-019-3615-4) contains supplementary material, which is available to authorized users.

## Background

*Fasciola gigantica* is considered the main liver fluke species responsible for fascioliasis in domestic animals and humans across vast land areas in tropical and subtropical regions. It causes great livestock economic losses and threatens human health [1, 2]. During migration and development in the definitive host, liver flukes are able to influence the immune microenvironment by continuously producing antigenic organisms, termed excretory/secretory products (ESPs). These ESPs are produced to diminish or even silence the host immune response, which maintains their survival [3–5]. Over the past decade, a number of studies have revealed that ESPs released by helminths could help these parasites evade host immune surveillance and clearance by affecting the maturation and immunofunctions of host dendritic cells (DCs). These are the most important innate immune cells that assume responsibility for presenting antigens [6, 7]. Studies have revealed that incubation with the ESPs of *F. gigantica* or *F. hepatica* (FgESPs or FhESPs) caused host antigen-presenting cells (APCs) to remain immature, and downregulated the expression of toll-like receptors (TLRs) *in vitro*, suggesting that some impaired mechanisms of the immune response might be involved [8–11].

Epigenetic modifications have been extensively studied, and the roles of these modifications in various biological processes, especially in the immunomodulatory process, have also been analyzed [12, 13]. These genetically heritable modifications of DNA that affect relevant proteins without changing the DNA sequence could be “memorized” by cells and maintained in subsequent cell proliferation [14]. DNA methylation is detected in almost all mammalian tissues and cell types. DNA methylation is catalyzed by a family of DNA methyltransferases (Dnmts) that form 5-methylcytosine (5-mC) [15, 16]. Facilitated by the ten-eleven translocation (TET) family proteins, 5-mC can be oxidized into 5-hydroxymethylcytosine (5hmC) [17]. This leads to the process of DNA hydroxymethylation, which is critical for the negative regulation of DNA methylation [18, 19]. Proper DNA hydroxymethylation facilitates the correct transcriptional regulation of gene expression by reducing the affinity between methylated DNA and the methyl-CpG-binding domain (MBD) of methylated cytosine-binding protein (MeCP) [18, 20]. It is important to note the key role of epigenetic control in defining myeloid cell polarization [21]. Changes in the balance between DNA methylation and DNA hydroxymethylation have been shown to be key for characterizing DC differentiation [22]. Furthermore, the development and functions of DCs may also be impaired by FgESPs. Thus, it is reasonable to assume that DNA (hydroxy)methylation might be involved in the process of DC development and functionalization impacted by FgESPs. Global DNA (hydroxy)methylation changes are shown to be important throughout the interaction between parasites-secreted ESPs components and immune cells [23]. However, little is known about the relationships among immune cell development, DNA (hydroxy)methylation and *Fasciola* spp. infection.

Here, we report findings from a genome-wide profiling of DNA (hydroxy)methylation in buffalo original DCs incubated with FgESPs *in vitro* based on a novel omics approach, (hydroxy)methylated DNA immunoprecipitation sequencing [(h)MeDIP-Seq]. The aim of this study was to examine whether FgESPs could disrupt the normal immune functions of DCs by altering the status of DNA (hydroxy)methylation.

## Methods

### Source of dendritic cells

To isolate DCs, one clinically healthy GX breed buffalo (3-years-old, female) was purchased from a local breeder in Nanning, Guangxi, China. The animal was managed under routine procedures, and provided commercial feed and clean water *ad libitum*. To avoid any possible pre-existing infection, antibiotics and anthelmintics were administered and a 4-week drug-withdrawal period was given, following the manufacturer’s instructions. After the clearance of drugs, the animal was confirmed as negative in terms of prior infection with common pathogens by negative fecal examination and negative blood smear examination prior to the start of the study.

### Antigen preparation and identification

Adult *F. gigantica* were obtained from the gall-bladder of one naturally infected buffalo slaughtered for human consumption at a local slaughterhouse (Nanning, Guangxi, China). The obtained parasite was confirmed as *F. gigantica* based on classical morphology and 100% similarity of the ITS2 sequence with that of the *F. gigantica* Guangxi isolate (GenBank: AJ557569) [24]. FgESPs were prepared as described previously [8]. Briefly, the collected flukes were brought to the laboratory and immediately placed in warm PBSG media (phosphate-buffered solution plus glucose and antibiotics) for 30 min in a CO_2_ incubator. Live flukes were collected, rinsed in fresh PBSG media (*c.*1 worm per 2 ml of PBSG) and incubated at 37 °C for 2 h. The supernatant was collected, centrifuged at 12,000×*g* for 30 min at 4 °C to remove the parasite eggs from the media and filtered through a 0.22 μm Millipore filter; the protein concentration was determined by a BCA kit (CW Biotech, Beijing, China). The sample was aliquoted for stored at −80 °C until use. Sodium dodecyl sulfate polyacrylamide gel electrophoresis (SDS-PAGE) and silver staining were used to describe the fraction profile of FgESPs. The prepared FgESPs possessing a highly similar SDS-PAGE pattern of protein fractions to the published reports [25] (shown in Additional file 1: Figure S1) were used for further experiments in the present study.

### Cell culture and antigen treatment

DCs derived from peripheral blood mononuclear cells (PBMCs) were obtained as previously described with slight modifications [26]. Briefly, PBMCs were isolated from heparinized blood samples of buffalo using a commercial lymphocyte separation medium (TBD, Tianjin, China) according to the manufacturer’s instructions. The cells were washed three times with PBS and resuspended in RPMI-1640 (Gibco, Grand Island, NY, USA) containing 10% fetal bovine serum (Gibco), 1% l-Glutamax (Gibco) and 1% PSN (Sigma, St. Louis, MO, USA). The PBMCs were allowed to adhere for 2 h at 37 °C in 5% CO_2_, the adherent cells were collected after washing three times with PBS. They were then cultured in complete culture medium containing GM-CSF (50 ng/ml; Kingfisher, St. Paul, MN, USA) and IL-4 (50 ng/ml, Kingfisher) for 6 days to induce DC differentiation. The harvested cells possessed a typical phenotype of mammal DCs (CD11c^high^ MHC II^high^ CD40^low^ CD80^low^ CD86^low^) confirmed by flow cytometric analysis using commercially available anti-bovine antibodies (Bio-Rad, Hercules, CA, USA). The selection of phenotypic markers was according to the reported DCs phenotypic features of bovines that are evolutionarily close to the buffalo [27, 28], since the phenotype of buffalo DCs remains inconclusive. 1 × 10^6^ cells per well with three replicate wells in the treatment group were treated with 200 μg/ml of FgESPs for 48 h, while cells treated with an equivalent volume of PBS served as the control group.

### Ultrastructural characterization

Scanning and transmission electron microscopes (SEM/TEM) were used to observe the ultrastructural characterization of DCs. After treatment with or without FgESPs for 48 h, the DCs were fixed in 3% glutaraldehyde, which was followed by 1% osmium tetroxide at 4 °C overnight. They were then dehydrated in a graded ethanol series (50, 70, 80, 90, 95 and 100%). For ultrastructural observation of the cell surface, cells were then processed in hexamethyldisilazane and air-dried. The samples were then photographed using a SEM (H-7650; Hitachi, Tokyo, Japan). To compare the intracellular structure, cells were embedded in epon resin, sliced into ultraslices on a Leica ultramicrotome (EM-UC7; Leica, Wetzlar, Germany) and stained with uranyl acetate and lead citrate. The intracellular structural changes of the samples were then observed using a TEM (S-3400N; Hitachi).

### DNA sample preparation

The genomic DNA (gDNA) of DCs used for (h)MeDIP and library construction was isolated from each individual sample using a DNeasy Blood & Tissue Kit (Qiagen, Redwood City, CA, USA). Quantification and quality assurance were evaluated using a NanoDrop 1000 (Thermo Fisher Scientific, Wilmington, DE, USA).

### (h)MeDIP

For (h)MeDIP, gDNA was sonicated to a size of ~200–500 bp with a Bioruptor sonicator (Diagenode, Philadelphia, PA, USA). About 1 μg of sonicated DNA was end-repaired, A-tailed and ligated to single-end adapters using a Genomic DNA Sample Prep Kit (Illumina, San Diego, CA, USA) according to the manufacturerʼs standard protocol. After unligated adapters were removed by size-selection with AMPure XP beads, the adaptor-ligated DNA was used for immunoprecipitation with a mouse monoclonal anti-5-methylcytosine antibody and a human monoclonal anti-5-hydroxymethylcytosine antibody (Diagenode). For this, DNA was heat-denatured at 94 °C for 10 min, then rapidly cooled on ice. Next, it was immunoprecipitated with 1 μl of primary antibody overnight at 4 °C with rocking agitation in 400 μl of immunoprecipitation buffer (0.5% BSA in PBS). As a negative control, non-specific mouse or human IgG immunoprecipitation was performed in parallel to the (hydroxy)methyl DNA immunoprecipitation (New England BioLabs, Ipswich, MA, USA). To recover the immunoprecipitated DNA fragments, 100 μl of protein G magnetic beads (Life Tech, Grand Island, NY, USA) were added and incubated for an additional 2 h at 4 °C with agitation. After immunoprecipitation, a total of five immunoprecipitation washes were performed with ice-cold immunoprecipitation buffer. Washed beads were resuspended in TE buffer with 0.25% SDS and 0.25 mg/ml proteinase K for 2 h at 65 °C and then allowed to cool down to room temperature.

### Library construction

To generate the library, the products of the (h)MeDIP, as well as the supernatant input DNA, were purified using Qiagen MinElute columns and eluted in 16 μl of elution buffer (Qiagen). Fourteen cycles of PCR were performed on 5 μl of the immunoprecipitated DNA using the single-end Illumina PCR primers. The resulting reactions were purified with Qiagen MinElute columns; then, a final size selection (~300–600 bp) was performed using AMPure XP beads. Quality control of the libraries was performed by an Agilent 2100 Bioanalyzer using an Agilent DNA 1000 Chip Kit. An aliquot of each library was diluted in the elution buffer to 5 ng/μl, and 1 μl was used in real-time PCR reactions to confirm enrichment of the (hydroxy)methylated region.

### Next-generation sequencing

The library was denatured with 0.1 M NaOH to generate single-stranded DNA. This was loaded onto channels of the flow cell at a concentration of 8 pM and amplified *in situ* using a HiSeq 3000/4000 PE Cluster Kit (Illumina). Sequencing was carried out by running 2 × 150 cycles on an Illumina HiSeq 4000 according to the manufacturer’s instructions.

### Data processing and analysis

The image analysis and base calling were performed using Off-Line Basecaller software (OLB v.1.8). After passing the Solexa CHASTITY quality filter, the clean reads were aligned to the genome of the family *Bubalus* (NCBI UMD_CASPUR_WB2_2.0) using HISAT2 software (v.2.1.0). (h)MeDIP-enriched regions (peaks) with statistical significance were identified for each sample by MACS v.2 (Model-based Analysis of ChIP-Seq) software (*P *< 10^−5^). (h)MeDIP-enriched regions (peaks) were annotated by the nearest gene using the newest NCBI database. Differentially (hydroxy)methylated regions [D(h)MRs] within the promoter region [−2.0 K to +2.0 K of transcriptional start site (TSS)] that reached statistical significance between the two groups were identified by diffReps (*P *< 0.05). Data from both the treated group and control group were normalized relative to the input samples.

### Functional enrichment analysis

Functional enrichment analysis was used to identify genes associated with ‘gain of 5-mC’ DMRs and ‘gain of 5-hmC’ DhMRs. The Gene Ontology (GO) project provides a controlled vocabulary of terms to describe gene and gene product characteristics (http://www.geneontology.org), and it covers three domains: biological process (BP), cellular component (CC) and molecular function (MF). We used Fisher’s exact test in the topGO package of Bioconductor to determine if there was more overlap between the differentially expressed (DE) list and the GO annotation list than would be expected by chance. The *P*-value produced by the topGO package denotes the significance of the enriched GO terms in the DE genes. Kyoto Encyclopedia of Genes and Genomes (KEGG) pathway analysis (http://www.kegg.jp/kegg/pathway.html) was used to suggest physiological functions of these genes and predict their associated signaling pathways.

### qRT-PCR

Total cellular RNA was isolated using the RNAiso Plus Kit (TaKaRa, Beijing, China) and reverse transcribed using the PrimerScript™ RT Reagent Kit (TaKaRa), following the manufacturer’s instructions. The mRNA expression of target genes was determined by quantitative reverse transcriptional PCR (qRT-PCR) using ChamQ SYBR Color qPCR Master Mix (Vazyme Biotech, Nanjing, China) and a CFX96 real-time PCR instrument (Bio-Rad). The target genes were cluster of differentiation (CD) 1a, CD40, CD80, CD83, CD86, major histocompatibility complex (MHC) II, TLR2, TLR4, interleukin (IL)-12B, tumor necrosis factor (TNF), transforming growth factor beta 1(TGF-β1) and TGF-β3. The mRNA level of each gene was normalized to the expression of bubaline glyceraldehyde-3-phosphate dehydrogenase (GAPDH), which served as the reference housekeeping gene. The 2^−*ΔΔCq*^ method was used to analyze the relative quantification of the target mRNA [29]. Primer sequences are listed in Table 1.

**Table 1:** List of primers used in the SYBR green-based qRT-PCR analysis

Gene	Primer sequence (5′–3′)	Gene	Primer sequence (5′–3′)
GAPDH-F	ACGTGTCTGTTGTGGATCTGAC	TLR2-F	CTGGGCTGTAATCATCCTGT
GAPDH-R	CGCTGTTGAAGTCGCAGGAG	TLR2-R	AGGTGATCTCGTTGTTGGAC
CD1a-F	TGTGCCACGTCTCAGGATT	TLR4-F	GGTTCAAACTTCGTGGGCTT
CD1a-R	TGTCCTGGTCTCCTAGACTGC	TLR4-R	AGCGGAGGTTTCTGAGTGAT
MHC II-F	CCTCGCTTGCCTGAATTTGC	CD40-F	GGGCTTTTGGATACCGTCTGT
MHC II-R	ACAGGTGCCGACTGATGC	CD40-R	AGCAGATGACACGTTGGAGAAG
IL-12B-F	CAGGGACATCATCAAACCAG	CD80-F	CCATCCTGCCTGGAAAAGTG
IL-12B-R	CTTGTGGCATGTGACTTTGG	CD80-R	GGTTATCGTTCATGTCAGTGATGGT
TGF-β1-F	CGTGCTAATGGTGGAATAC	CD83-F	TGTGAAGCCCTTTGCTTGAC
TGF-β1-R	GCCAGGAATTGTTGCTATA	CD83-R	CCAAGAGGTTGACCAGATAG
TGF-β3-F	ACCTGGGATTAAGGACAAG	CD86-F	AGAAGGTCCCAAGGACTGGT
TGF-β3-R	TCAAGGAAGAAGGCGAGA	CD86-R	GCTTGGCACAGGTGACTTTG
TNF-F	TAACAAGCCGGTAGCCCACG	TNF-R	GCAAGGGCTCTTGATGGCAGA

### Statistical analysis

All data are expressed as the mean ± SEM of three independent experiments. The mRNA expression levels of target genes in the treated and untreated groups were compared. Statistical comparisons and graphing were performed by GraphPad Prism v.6.02 (GraphPad, San Diego, CA, USA) using an unpaired Student’s t*-*test. The level of significance for all analyses was evaluated with a confidence interval > 95% (*P *< 0.05).

## Results

### DC morphological and phenotypic changes

We first examined the morphology of buffalo-derived DCs treated with or without FgESPs. The ultrastructure of DCs was determined using SEM and TEM (Additional file 2: Figure S2). The control DCs had varying lengths of little tails or well-developed dendritic synapses on their surface, which were rarely or not observed in FgESPs-treated DCs. Compared to the control DCs, the number of lysosomes and phagosome-like balls were visibly increased in most of DCs treated with FgESPs. Moreover, the expression of DC maturation-associated molecular markers was measured by qRT-PCR, demonstrating a general pattern of decreased expression for CD1a, CD40, CD83, CD86 and MHC II in FgESPs-treated DCs, but not for CD80 (Additional file 3: Figure S3). Among these, CD1a (*t*_(2)_ = 5.390, *P* = 0.0327) and MHC II (*t*_(2)_ = 8.412, *P* = 0.0138) mRNA levels were decreased significantly compared to the controls.

### Global changes in DNA (hydroxy)methylation

**Fig. 1:**
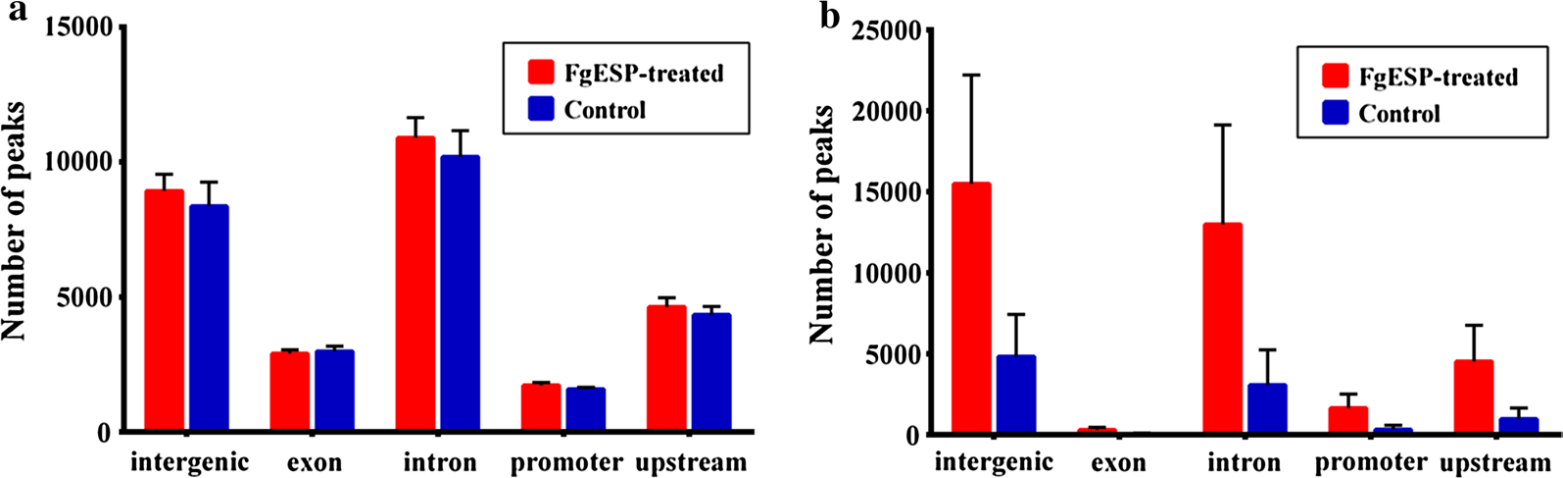
The distribution of 5-mC (**a**) and 5-hmC (**b**) peaks in genome components. The x-axis represents different regions of the genome, and the y-axis represents the number of peaks. Green bars represent the peaks of the control DCs, and blue bars represent the peaks of the FgESPs-treated DCs

**Fig. 2:**
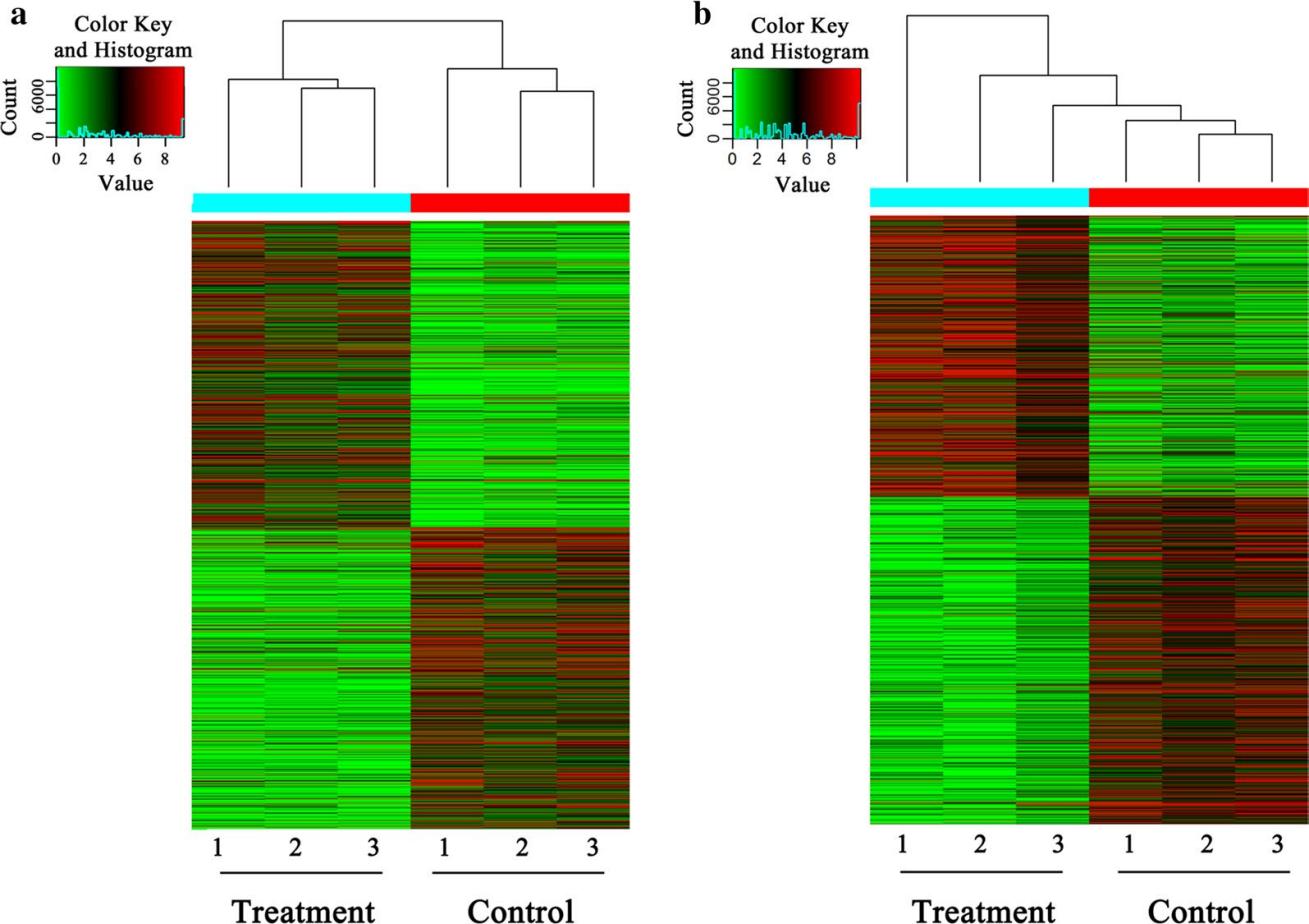
Heatmap of differential 5-mC (**a**) and 5-hmC (**b**) regions (peaks) showing the most significant differences between FgESPs-treated DCs (treatment) and untreated DCs (control). Green regions represent downregulation of peaks; red regions represent upregulation of peaks

We extracted total gDNA from the treated DCs and the control DCs. The genome-wide 5-mC and 5-hmC profiles were determined by (h)MeDIP-seq. Reads of the sequencing data for 5-mC and 5-hmC that were identified by the MACS approach are listed in Table 2. The aligned percentage of control-input was 76.16% and the treatment-input was 71.16%. The distribution of 5-mC and 5-hmC peaks for each of the 5 genomic regions (intergenic, exon, intron, promoter, upstream 2k) is shown in Fig. 1. The number of 5-mC peaks across all genome components was about the same for both groups. However, FgESPs-treated DCs had a higher number of 5-hmC peaks than the control DCs. The number of differential 5-mC and 5-hmC peaks between the two groups is shown in Table 3. The number of 5-mC was much higher than that of 5-hmC. The peaks differentially enriched in 5-mC and 5-hmC showed different clustering patterns between treated DCs and control DCs (Fig. 2). This indicates that there were significant changes in methylation and hydroxymethylation. It also indicates that, unlike that of 5-hmC, the 5-mC-enriched regions of the two groups were divided into two distinct topological branches.

**Table 2:** Number of reads generated by (h)MeDIP-Seq for each sample

	Control 1	Control 2	Control 3	Control-input	Treatment 1	Treatment 2	Treatment 3	Treatment-input
MeDIP-Seq								
Total no. of aligned reads	9,596,656	10,154,458	8,820,009	15,171,722	9,420,181	10,596,590	10,972,759	16,743,375
Aligned percentage	49.49	50.39	46.60	76.16	46.05	47.18	50.82	71.66
(h)MeDIP-Seq								
Total no. of aligned reads	21,561,276	14,073,616	9,713,113	15,171,719	10,212,535	11,525,320	23,343,030	16,743,373
Aligned percentage	70.77	68.95	63.40	76.16	57.22	64.09	42.82	71.66

**Table 3:** Number of differential 5-mC and 5-hmC peaks

	5-mC-TvC	5-mC-CvT	5-hmC-TvC	5-hmC-CvT
Peak	112,920	111,534	3605	4206
Peak promoter	4864	4687	295	276

*Abbreviations*: TvC, upregulated peaks in FgESPs-treated DCs *vs* untreated control DCs; CvT, downregulated peaks in FgESPs-treated DCs *vs* untreated control DCs

### D(h)MR-associated genes of the promoter region

Recent studies have shown that DNA methylation or demethylation in the gene promoter region plays a crucial role in gene expression. Here, we further investigated this role by conducting bioinformatic analysis of D(h)MRs in the promoter region to obtain site-specific D(h)MRs in both groups. The statistically significant DMRs (fold changes >2-fold, *P* < 0.05) in the promoter region included 8392 RefSeq IDs. There were 2752 genes that had hypermethylation (5-mC-TvC) in the promoter region and 2680 genes that had hypomethylation (5-mC-CvT). The statistically significant DhMRs (fold changes >2-fold, *P* < 0.05) in the promoter regions included 501 RefSeq IDs. Hydroxymethylation was upregulated (5-hmC-TvC) in 182 genes and downregulated (5-hmC-CvT) in 178 genes (Table 4).

**Table 4:** Number of D(h)MR-associated genes in promoter regions

	5-mC-TvC	5-mC-CvT	5-hmC-TvC	5-hmC-CvT
Number	2752	2680	182	178

*Abbreviations*: TvC, upregulated genes in FgESPs-treated DCs *vs* untreated control DCs; CvT, downregulated genes FgESPs-treated DCs *vs* untreated control DCs

### Functional enrichment analysis

**Fig. 3:**
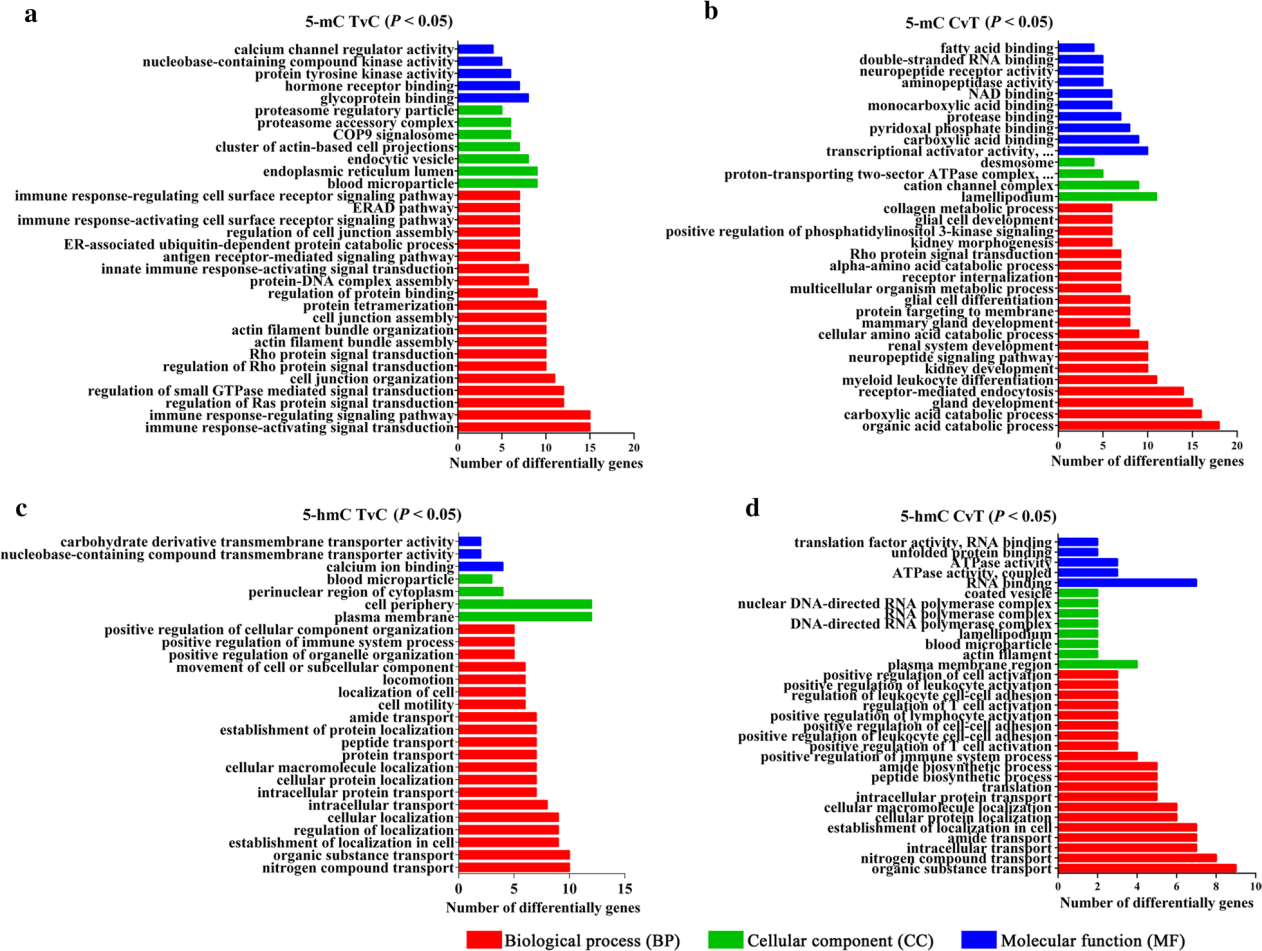
The significant GO categories of 5-mC and 5-hmC genes. Red, green and blue columns represent biological processes (BP), cellular component (CC) and molecular function (MF), respectively. **a** Upregulated methylation terms in FgESPs-treated DCs *vs* control DCs (TvC). **b** Downregulated methylation terms in FgESPs-treated DCs *vs* control DCs (CvT). **c** Upregulated hydroxymethylation terms in FgESPs-treated DCs *vs* control DCs (TvC). **d** Downregulated hydroxymethylation terms FgESPs-treated DCs *vs* control DCs (CvT)

The significant GO terms associated with 5-mC genes are shown in Fig. 3a, b (*P* < 0.05). The 5-mC-TvC genes primarily occurred in the immune response-related biological processes which were with our particular interests, including ‘regulation of interleukin-6 production’ (*P* = 0.02267), ‘immune response-regulating cell surface receptor signaling pathway’ (*P* = 0.0183), and etc. The 5-mC-CvT genes were enriched in the categories of ‘metabolism’ and ‘binding’, including ‘multicellular organism metabolic process’ (*P* = 0.0101), ‘protease binding’ (*P* = 0.0194), and etc. The KEGG pathway analysis showed that significantly hypermethylated genes were enriched in 16 pathways. Among these, ‘proteoglycans in cancer’ (*P* = 0.0040) and ‘NF-kappa B signaling pathway’ (*P* = 0.0316), involving TLR2, TLR4 and IL-12B genes, were our main focus due to their roles in immunity response. A total of 23 pathways were found to be enriched by hypomethylated genes. For instance, the hypomethylated gene TGF-β3 contained in the ‘TGF-beta signaling pathway’ (*P* = 0.0011) was also enriched in several other pathways. All significant pathways (*P* < 0.05) are listed in Additional file 4: Table S1.

The significant GO terms associated with 5-hmC genes of the DhMR are shown in Fig. 3c, d (*P* < 0.05). The 5-hmC-TvC genes were primarily enriched in transport-related biological processes, including ‘organic substance transport’ (*P* = 0.0105), ‘nitrogen compound transport’ (*P* = 0.0033), and etc. The 5-hmC-CvT genes were enriched in the categories of ‘regulation’ including ‘positive regulation of T cell activation’ (*P* = 0.0022), ‘positive regulation of lymphocyte activation’ (*P* = 0.0048), and etc. As listed in Additional file 4: Table S2, our KEGG pathway analysis showed 20 pathways enriched by high hydroxymethylated genes, 10 of which contained the TNF gene. Meanwhile, 17 pathways were enriched by low hydroxymethylated genes (*P* < 0.05).

**Fig. 4:**
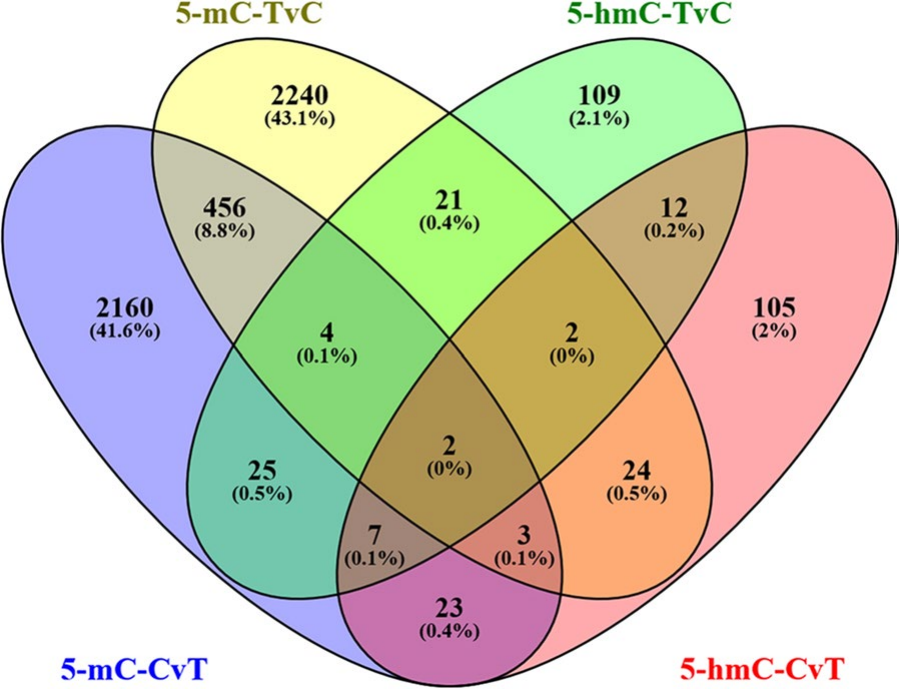
Venn diagram of 5-mC and 5-hmC genes in the promoter region. A total of 2680 genes had hypomethylation (5-mC-CvT), and 2752 genes had hypermethylation (5-mC-TvC). There were 182 upregulated 5-hmC genes (5-hmC-TvC) and 178 downregulated 5-hmC genes (5-hmC-CvT). A total of 111 genes underwent both 5-mC and 5-hmC changes by cross-alignment

**Fig. 5:**
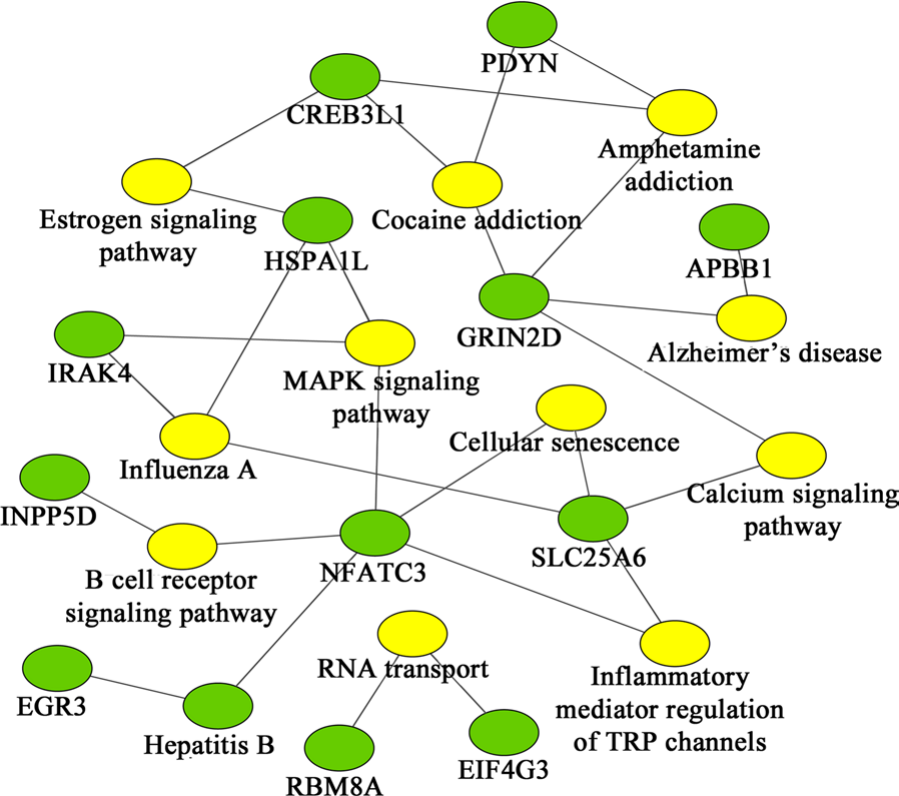
Pathway analysis of significant changes in both 5-mC and 5-hmC levels. Green hexagons represent genes; yellow hexagons represent pathways

A total of 111 genes that underwent both 5-mC and 5-hmC changes were found by cross-alignment in this study (Fig. 4). As shown in Fig. 5, there were 12 major enriched pathways including ‘MAPK signaling pathway’, ‘inflammatory mediator regulation of TRP channels’ and ‘Hepatitis B’, etc. The network included 12 interconnected and enriched genes: PDYN, IRAK4, HSPA1L, GRIN2D, SLC25A6, NFATC3, INPP5D, CREB3L1, APBB1, EGR3, RBM8A and EIF4G3.

### Changes in mRNA level of genes associated with innate immunity

**Fig. 6:**
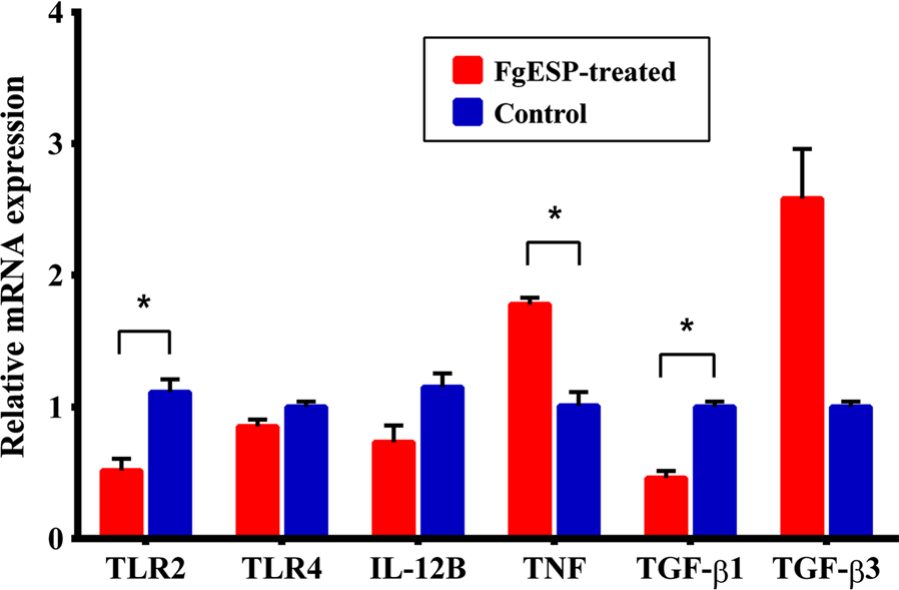
Changes in mRNA expression of TLR2, TLR4 and key cytokines in DCs treated with FgESPs. The x-axis represents the names of genes and the y-axis represents the relative mRNA expression of target genes. Expression is relative to the buffalo GAPDH gene based on the 2^−*ΔΔCq*^ calculation. Red bars represent the FgESPs-treated group and blue bars represent the control group. Columns show the means and error bars show SEMs. Significant differences were compared to the control group. *P < 0.05, **P < 0.01 (Student’s t-test)

**Fig. 7:**
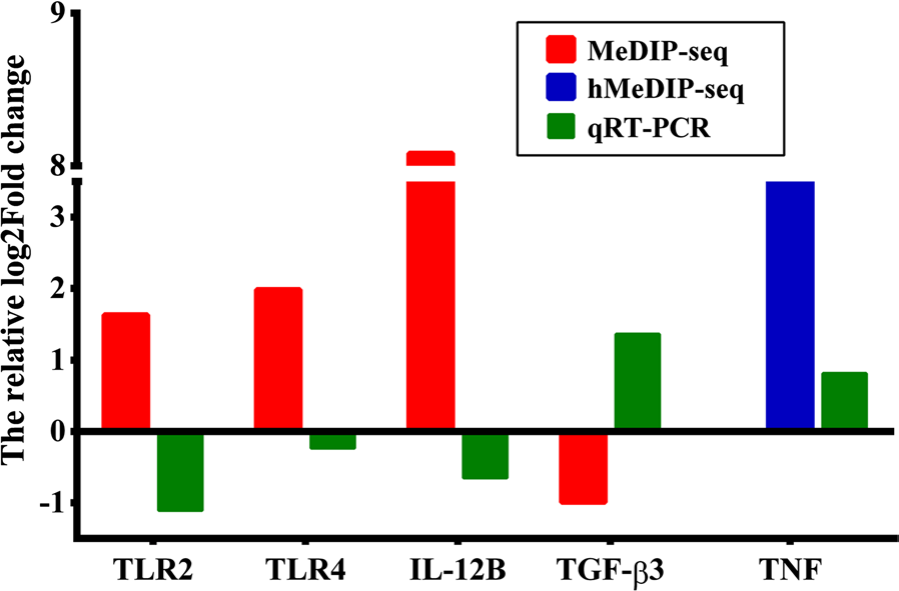
Comparative sketch of the relative fold changes between (h)MeDIP-seq and qRT-PCR. The relative fold change of (h)MeDIP-seq and qRT-PCR was calculated based on Log2. Red, blue and green bars represent MeDIP-seq, hMeDIP-seq and qRT-PCR analyses, respectively. The x-axis indicates the names of the genes and the y-axis represents the relative Log2 fold changes of the target genes. This figure indicates that the results obtained from qRT-PCR matched the prediction of the (h)MeDIP-seq analyses

We next determined if FgESPs actually altered DC maturation and immune functions, by assessing mRNA expression changes for 6 genes associated with innate immunity (TLR2, TLR4, IL-12B, TNF, TGF-β1 and TGF-β3) in buffalo DCs treated with or without FgESPs (Fig. 6). The mRNA levels of TGF-β3 (*t*_(2)_ = 4.249, *P* = 0.0039) and TNF (*t*_(2)_ = 5.048, *P* = 0.0371) in treated DCs were significantly higher than the control DCs, while the levels of TLR2 (*t*_(2)_ = 4.541, *P* = 0.0452) and TGF-β1 (*t*_(2)_ = 6.931, *P* = 0.0202) were significantly lower. The mRNA levels of both IL-12B and TLR4 in treated DCs displayed a very slight decline, although non-significant (*t*_(2)_ = 3.094, *P* = 0.0905 for IL-12B; *t*_(2)_ = 2.048, *P* = 0.1771 for TLR4). The pattern of gene transcriptional profile was consistent with that of sequencing analyses, confirming the reliability of the (h)MeDIP-Seq approach (Fig. 7).

## Discussion

The dynamic balance between DNA methylation and hydroxymethylation that regulates gene expression in mammalian cells is key to elucidating the underlying molecular mechanisms of DC maturation and function. Furthermore, DNA (hydroxy)methylation changes occur throughout the transition from monocytes to mature DCs. This may alter the expression of cytokines and other corresponding genes, therefore influencing DC differentiation and development [30, 31]. In the present study, we observed an increase of intracellular lysosomes and phagosome-like balls but fewer and less-developed synapses, associated with a downregulated gene expression profile involved in DC maturation. This reflects a possibly suppressive effect of FgESPs on development and functions of buffalo DCs *in vitro*. We therefore sequenced and analyzed the DNA (hydroxy)methylation based on a (h)MeDIP-Seq approach. To the best of our knowledge, this is the first analysis on the genome-wide profiling of DNA (hydroxyl)methylation for buffalo DCs challenged with FgESPs.

Methylation and demethylation of DNA promotors decide the fate of gene expression, playing an important role in various biological processes [32–34]. In the present study, GO analysis of 2752 5-mC-TvC genes in the promoter region indicated that these genes primarily occurred in ‘regulation’ and ‘immune response’-related biological processes, while 2680 hypomethylated genes were enriched in the categories of ‘metabolism’ and ‘binding’. Although DNA methylation is known to be associated with the suppression of gene transcription, this is not a completely fixed rule. A theory was proposed by Jones & Gonzalgo [35, 36] that DNA methylation occurring at the site of promotor regions could alter the reactions between proteins and DNA that change the structure of chromatin or cause changes the transcriptional level. In our study, hypermethylated genes were enriched in ‘protein-DNA complex assembly’ (*P* = 0.0282), which corroborates this theory. Moreover, outcomes of gene transcription can also be determined by the location of the methylation changes relative to the transcription start site [37].

5-hmC peaks were mainly enriched in the intergenic and intron regions of the gene and were not evenly distributed throughout the genome in buffalo DCs, which is in agreement with a previous study in human cells [38]. GO analysis showed that both upregulated and downregulated hydroxymethylated genes in the FgESPs-treated buffalo DCs were enriched in the same pathways, such as ‘nitrogen compound transport’ (*P* = 6.08e−05) and ‘cellular protein localization’ (*P* = 0.0011). Although several previous studies have illustrated that hydroxymethylation might be correlated with transcriptional activation in eukaryotes, the precise biochemical mechanisms remain unclear [39, 40]. Our GO analysis showed that hydroxymethylated genes and hypomethylated genes were enriched in different categories. These results are insufficient to discuss the relationship between DNA hydroxymethylation and transcriptional activation in this study, which currently has no unified theory.

KEGG pathway analysis identified several pathways involved in immune response, receptor signaling, cancers and some diseases. Among these, immune response-related pathways have caught our particular interest. The pronounced DNA hypermethylation of TLR2, TLR4 and IL-12B (the most functional unit of IL-12), and the high level of hydroxymethylation of TNF in treated DCs were enriched in multiple pathways. Studies have shown that TLR4-dependent IL-12 productions play crucial roles in promoting DC maturation and the pro-inflammatory immune response to eliminate pathogens from the infection [41]. TNF, which can be mediated by both TLR2-dependent and TLR2-independent pathways [42–44], is known to induce a semi-mature phenotype and a limited activation of DCs [45]. In agreement with the prediction of (h)MeDIP-Seq, our qRT-PCR analysis showed that the mRNA levels of TLR2, TLR4 and IL-12B were downregulated, and TNF transcription was increased. This may contribute to the suppression of buffalo DC maturation. It is known that liver fluke infection utilizes their ESPs to weaken immune cells and reduce the Th1-type response through disrupting TLR signaling [46]. *Fasciola gigantica* infection in buffaloes has shown to suppress the TLR4 signaling pathway [47, 48]. Similarly, the expression of TLR2 and TLR4 in immune cells could be suppressed by ESPs of *F. hepatica* [9, 10, 49]. In addition, the NF-κB signaling pathway enriched by several hypermethylated genes (including TLR4) may indicate a possible suppression of NF-κB activation that also mediates by TLR signaling. Collectively, these findings imply a possible mechanism of FgESPs in suppressing TLR recognition or TLR signaling cascade that influences DC maturation and functions.

Hypomethylation of TGF-β3 was enriched in the ‘TGF-beta signaling pathway’, which has been shown to be influenced by *F. gigantica* infection in buffaloes [48]. qRT-PCR also confirmed that the mRNA level of TGF-β3 was increased, while TGF-β1 expression was decreased in the treated DCs. Both TGF-β1 and TGF-β3 isoforms are able to induce the activation of Th17 cells that negatively regulate the differentiation of Th1 or Th2 cells, but with different manners [50–52]. To our knowledge, this is the first demonstration that TGF-β1 and TGF -β3 have opposite expression trends in buffaloes, which is similar to the trends observed in humans [53]. However, it is surprising that our analysis did not find any change of methylation of TGF-β1. Moreover, as competitors, TGF-β1 and TNF always play opposite roles in the development of DCs [54]. The regulatory pattern of decreased TGF-β1 but increased TNF observed in our study may largely contribute to the suppression of DC maturation.

It is noteworthy that a total of 111 genes had changes in both methylation and hydroxymethylation in the present study. Pathway analysis showed that there were 12 enriched pathways including 12 major enriched genes. Among these, NFATC3 (nuclear factor of activated T cells 3) is a member of the NFAT family of transcription factors, and is necessary for gene transcription in immune cells. Through mediation by IRF7 (interferon regulatory factors), NFATC3 may enhance IFN expression in DCs [55]. IRAK4 (interleukin-1 receptor-associated kinase 4) also plays a key role in the secretion of IFN- α/β and -γ by DCs [56]. Hence, changes in 5-mC and 5-hmC in NFATC3 and IRAK4 may affect the ability of DCs to secrete IFN, and therefore disrupt the balance of Th1/Th2 response. Studies focusing on the other ten genes may provide more clues.

In addition to immune response-related pathways, we also observed enrichment in certain cancer-related pathways, such as ‘proteoglycans in cancer’, ‘pathways in cancer’ and ‘bladder cancer’. Genes with 5-mC and 5-hmC level changes enriched in these pathways, such as BMP4, HSP90AA1, DAPK3, FADD and CASP8, have been already reported as potential epigenetic biomarkers for liver cancer [57–59]. There are often liver complications associated with the reported cases of fascioliasis infection, including liver fibrosis, cirrhosis and possibly cancer [60, 61]. However, there are a very limited number of reports on chronic infection with fascioliasis, and there is a lack of follow-up studies. Thus, it remains to be confirmed if it can lead to cancer. What is clear though, is that repeated infection of *F. gigantica* is a dynamic process of inflammatory and anti-inflammatory responses. Pro-inflammatory cytokines, such as IL-6 [62], TNF-α [63] and IL-12 [64], contribute to chronic inflammation. They also appear to influence the progression of carcinogenesis [65, 66]. Chronic inflammation is currently considered a critical determinant of the initiation and progression of various forms of cancer, and the role of NF-κB is crucial to linking chronic inflammation and cancer [67, 68]. In addition, many studies have shown that worms that live in the liver can induce cancer. Chronic infections of human liver flukes, such as *Clonorchis sinensis*, *Opisthorchis viverrini* and *Opisthorchis felineus*, can cause liver cancer or cholangiocarcinoma [69–71]. Pro-inflammatory cytokines that contribute to the clearance of parasites appear to be dysregulated when the liver flukes become chronic infections [72–74]. Thus, we speculate that there may be a potential carcinogenic risk for hosts infected by *F. gigantica*. However, this theory needs further confirmation by conducting animal experiments and in-depth epidemiological investigations.

## Conclusions

In summary, we discovered that FgESPs could induce higher levels of DNA methylation rather than hydroxymethylation in the promoter regions of DCs. Epigenetic changes may be one possible mechanism of FgESPs-mediated immune tolerance in DCs. The study provides insight into the gene regulation network specific to FgESPs in host DCs.

## Additional files

**Additional file 1: Figure S1.** Molecular weight and protein profile of FgESPs in SDS-PAGE gel. Lane 1: molecular size marker ranging from 10 to 180 kDa (Thermo Fisher Scientific, Wilmington, DE, USA); Lane 2:5 μg of prepared FgESPs.

**Additional file 2: Figure S2.** Ultrastructural changes of buffalo DCs treated with FgESPs. Cell surface and intracellular ultrastructural features of DCs were observed using scanning electron microscopy (SEM) and transmission electron microscopy (TEM), respectively. The results demonstrate the occurrence of several intracellular lysosomes (solid box) and phagosome-like balls (dashed box), as well as fewer synapses (arrows) in DCs treated with FgESPs (**c**, **d**) rather than in the control DCs (**a**, **b**). *Scale-bars*: **a**, **c**, 4 μm; **b**, **d**, 1 μm.

**Additional file 3: Figure S3.** Changes in mRNA expression of surface markers in buffalo DCs treated with FgESPs. The x-axis indicates the names of genes and the y-axis represents the relative mRNA expression of target genes. Expression is relative to the buffalo GAPDH gene based on the 2^−ΔΔCq^ calculation. Red bars represent the FgESPs-treated groups and blue bars represent the control DCs. Columns show the means, and error bars show SEMs. Significant differences were compared to the control group. *P < 0.05 (Student’s t-test).

**Additional file 4: Table S1.** Pathway analysis of DMR-associated genes. **Table S2.** Pathway analysis of DhMR-associated genes.

## Data Availability

The datasets supporting the findings of this article are included within the article. The (h)MeDIP-Seq raw data are available in the NCBI GEO repository under accession numbers GSE125734 and GSE125735.

## Abbreviations

FgESP: *Fasciola gigantica* excretory/secretory products
DCs: dendritic cells
PBMCs: peripheral blood mononuclear cells
5-mC: 5-methylcytosine
5-hmC: 5-hydroxymethylcytosine
(h)MeDIP-Seq: (hydroxy)methylated DNA immunoprecipitation sequencing
GO: Gene Ontology
Dnmts: DNA methyltransferases
TET: ten-eleven translocation
MeCP: methylated cytosine-binding protein
MBD: methyl-CpG-binding domain
PBS: phosphate-buffered solution
PBSG: phosphate-buffered solution plus glucose
PCR: polymerase chain reaction
MACS: model-based analysis of ChIP-Seq
D(h)MRs: differentially (hydroxy)methylated regions
TSS: transcriptional start site
qRT-PCR: quantitative reverse transcriptase real-time PCR
BP: biological process
CC: cellular component
MF: molecular function
KEGG: Kyoto Encyclopedia of Genes and Genomes
DE: differentially expressed
GAPDH: glyceraldehyde-3-phosphate dehydrogenase
Cq: quantification cycle
TLR: toll-like receptors
TNF: tumor necrosis factor
TGF: transforming growth factor
IL: interleukin
LPS: lipopolysaccharide
NFATC3: nuclear factor of activated T cells 3
IRAK4: interleukin-1 receptor-associated kinase 4

## References

[1] Chen JX, Chen MX, Ai L, Xu XN, Jiao JM, Zhu TJ (2013). An outbreak of human fascioliasis gigantica in southwest China. PLoS ONE.

[2] Lin RQ, Dong SJ, Nie K, Wang CR, Song HQ, Li AX (2007). Sequence analysis of the first internal transcribed spacer of rDNA supports the existence of the intermediate *Fasciola* between *F. hepatica* and *F. gigantica* in mainland China. Parasitol Res.

[3] Garza-Cuartero L, OʼSullivan J, Blanco A, McNair J, Welsh M, Flynn RJ (2016). *Fasciola hepatica* infection reduces *Mycobacterium bovis* burden and mycobacterial uptake and suppresses the pro-inflammatory response. Parasite Immunol.

[4] Cervi L, Serradell MC, Guasconi L, Masih DT (2009). New insights into the modulation of immune response by *Fasciola hepatica* excretory-secretory products. Curr Immunol Rev.

[5] Rodríguez E, Noya V, Cervi L, Chiribao ML, Brossard N, Chiale C (2015). Glycans from *Fasciola hepatica* modulate the host immune response and TLR-induced maturation of dendritic cells. PLoS Negl Trop Dis.

[6] Hamilton CM, Dowling DJ, Loscher CE, Morphew RM, Brophy PM, OʼNeill SM (2009). The *Fasciola hepatica* tegumental antigen suppresses dendritic cell maturation and function. Infect Immun.

[7] Walsh KP, Brady MT, Finlay CM, Boon L, Mills KH (2009). Infection with a helminth parasite attenuates autoimmunity through TGF-beta-mediated suppression of Th17 and Th1 responses. J Immunol.

[8] Falcón C, Carranza F, Martínez FF, Knubel CP, Masih DT, Motrán CC (2010). Excretory-secretory products (ESP) from *Fasciola hepatica* induce tolerogenic properties in myeloid dendritic cells. Vet Immunol Immunopathol.

[9] Flynn RJ, Mulcahy G (2008). Possible role for Toll-like receptors in interaction of *Fasciola hepatica* excretory/secretory products with bovine macrophages. Infect Immun.

[10] Martin I, Cabán-Hernández K, Figueroa-Santiago O, Espino AM (2015). *Fasciola hepatica* fatty acid-binding protein inhibits TLR4 activation and suppresses the inflammatory cytokines induced by lipopolysaccharide *in vitro* and *in vivo*. J Immunol.

[11] Dowling DJ, Hamilton CM, Donnelly S, La Course J, Brophy PM, Dalton J (2010). Major secretory antigens of the helminth *Fasciola hepatica* activate a suppressive dendritic cell phenotype that attenuates Th17 cells but fails to activate Th2 immune responses. Infect Immun.

[12] Li H, Hong G, Xu H, Guo Z (2015). Application of the rank-based method to DNA methylation for cancer diagnosis. Gene.

[13] Voorde LVD, Speeckaert R, Gestel DV, Bracke M, Neve WD, Delanghe J (2012). DNA methylation-based biomarkers in serum of patients with breast cancer. Mutat Res.

[14] Goldberg AD, Allis CD, Bernstein E (2007). Epigenetics: a landscape takes shape. Cell.

[15] Globisch D, Münzel M, Müller M, Michalakis S, Wagner M, Koch S (2010). Tissue distribution of 5-hydroxymethylcytosine and search for active demethylation intermediates. PLoS ONE.

[16] Ruzov A, Tsenkina Y, Serio A, Dudnakova T, Fletcher J, Bai Y (2011). Lineage-specific distribution of high levels of genomic 5-hydroxymethylcytosine in mammalian development. Cell Res.

[17] Tahiliani M, Koh KP, Shen Y, Pastor WA, Bandukwala H, Brudno Y (2009). Conversion of 5-methylcytosine to 5-hydroxymethylcytosine in mammalian DNA by MLL partner TET1. Science.

[18] Dahl C, Grønbæk K, Guldberg P (2011). Advances in DNA methylation: 5-hydroxymethylcytosine revisited. Clin Chim Acta.

[19] Branco MR, Ficz G, Reik W (2011). Uncovering the role of 5-hydroxymethylcytosine in the epigenome. Nat Rev Genet.

[20] Illingworth RS, Bird AP (2009). CpG islands—‛a rough guideʼ. FEBS Lett.

[21] Álvarez-Errico D, Vento-Tormo R, Sieweke M, Ballestar E (2015). Epigenetic control of myeloid cell differentiation, identity and function. Nat Rev Immunol.

[22] Zawada AM, Schneider JS, Michel AI, Rogacev KS, Hummel B, Krezdorn N (2016). DNA methylation profiling reveals differences in the 3 human monocyte subsets and identifies uremia to induce DNA methylation changes during differentiation. Epigenetics.

[23] Deaton AM, Cook PC, De Sousa D, Phythian-Adams AT, Bird A, MacDonald AS (2014). A unique DNA methylation signature defines a population of IFN-γ/IL-4 double-positive T cells during helminth infection. Eur J Immunol.

[24] Huang WY, He B, Wang CR, Zhu XQ (2004). Characterisation of *Fasciola species* from mainland China by ITS-2 ribosomal DNA sequence. Vet Parasitol.

[25] Ridi EL, Salah M, Wagih A, William H, Tallima H, Shafie EL (2007). *Fasciola gigantica* excretory-secretory products for immunodiagnosis and prevention of sheep fasciolosis. Vet Parasitol.

[26] Pinchuk LM, Boyd BL, Kruger EF, Roditi I, Furger A (2003). Bovine dendritic cells generated from monocytes and bone marrow progenitors regulate immunoglobulin production in peripheral blood B cells. Comp Immunol Microbiol Infect Dis.

[27] Seo KS, Park JY, Davis WC, Fox LK, Mcguore MA, Park YH (2009). Superantigen-mediated differentiation of bovine monocytes into dendritic cells. J Leukocyte Biol.

[28] Miyazawa K, Aso H, Honda M, Kido T, Minashima T, Kanaya T (2006). Identification of bovine dendritic cell phenotype from bovine peripheral blood. Res Vet Sci.

[29] Schmittgen TD, Livak KJ (2008). Analyzing real-time PCR data by the comparative C_T_ method. Nat Protoc.

[30] Bullwinkel J, Lüdemann A, Debarry J, Singh PB (2011). Epigenotype switching at the CD14 and CD209 genes during differentiation of human monocytes to dendritic cells. Epigenetics.

[31] Zhang X, Ulm A, Somineni HK, Oh S, Weirauch MT, Zhang HX (2014). DNA methylation dynamics during *ex vivo* differentiation and maturation of human dendritic cells. Epigenetics Chromatin.

[32] Shen J, Wang S, Zhang YJ, Kappil MA, Chen WuH, Kibriya MG (2012). Genome-wide aberrant DNA methylation of microRNA host genes in hepatocellular carcinoma. Epigenetics.

[33] Jones PA, Baylin SB (2002). The fundamental role of epigenetic events in cancer. Nat Rev Genet.

[34] Jones PA, Laird PW (1999). Cancer epigenetics comes of age. Nat Genet.

[35] Jones PL, Veenstra GJ, Wade PA, Vermaak D, Kass SU, Landsberger N (1998). Methylated DNA and MeCP2 recruit histone deacetylase to repress transcription. Nat Genet.

[36] Gonzalgo ML, Hayashida T, Bender CM, Pao MM, Tsai YC, Gonzales FA (1998). The role of DNA methylation in expression of the p19/p16 locus in human bladder cancer cell lines. Cancer Res.

[37] Jones PA, Takai D (2001). The role of DNA methylation in mammalian epigenetics. Science.

[38] Stroud H, Feng S, Morey Kinney S, Pradhan S, Jacobsen SE (2011). 5-Hydroxymethylcytosine is associated with enhancers and gene bodies in human embryonic stem cells. Genome Biol.

[39] Ficz G, Branco MR, Seisenberger S, Santos F, Krueger F, Hore TA (2011). Dynamic regulation of 5-hydroxymethylcytosine in mouse ES cells and during differentiation. Nature.

[40] Ito S, D’Alessio AC, Taranova OV, Hong K, Sowers LC, Zhang Y (2010). Role of Tet proteins in 5mC to 5hmC conversion, ES-cell self-renewal and inner cell mass specification. Nature.

[41] Durães FV, Carvalho NB, Melo TT, Oliveira SC, Fonseca CT (2009). IL-12 and TNF-α production by dendritic cells stimulated with *Schistosoma mansoni* schistosomula tegument is TLR4- and MyD88-dependent. Immunol Lett.

[42] Weiss S, Levy H, Fisher M, Kobiler D, Altboum Z (2009). Involvement of TLR2 in innate response to *Bacillus anthracis* infection. Innate Immun.

[43] Takada H, Uehara A (2006). Enhancement of TLR-mediated innate immune responses by peptidoglycans through NOD signaling. Curr Pharm Des.

[44] Reske A, Pollara G, Krummenacher C, Katz DR, Chain BM (2008). Glycoprotein-dependent and TLR2-independent innate immune recognition of herpes simplex virus-1 by dendritic cells. J Immunol.

[45] Pletinckx K, Stijlemans B, Pavlovic V, Laube R, Brandl C, Kneita S (2011). Similar inflammatory DC maturation signatures induced by TNF or *Trypanosoma brucei* antigens instruct default Th2-cell responses. Eur J Immunol.

[46] Cwiklinski K, O’Neill SM, Donnelly S, Dalton JP (2016). A prospective view of animal and human Fasciolosis. Parasite Immunol.

[47] Zhang FK, Hou JL, Guo AJ, Tian AL, Sheng ZA, Zheng WB (2018). Expression profiles of genes involved in TLRs and NLRs signaling pathways of water buffaloes infected with *Fasciola gigantica*. Mol Immunol.

[48] Zhang FK, Zhang XX, Elsheikha HM, He JJ, Zhu XQ (2017). Transcriptomic responses of water buffalo liver to infection with the digenetic fluke *Fasciola gigantica*. Parasites Vectors.

[49] Donnelly S, O’Neill SM, Sack CM, Robinson MW, Turnbull L, Whitchurch C (2010). Helminth cysteine proteases inhibit TRIF-dependent activation of macrophages via degradation of TLR3. J Biol Chem.

[50] Bettelli E, Carrier Y, Gao W, Korn T, Strom TB, Oukka M (2006). Reciprocal developmental pathways for the generation of pathogenic effector TH17 and regulatory T cells. Nature.

[51] Zhou L, Ivanov II, Spolski R, Min R, Shenderov K, Egawa T (2007). IL-6 programs TH-17 cell differentiation by promoting the sequential engagement of the IL-21 and IL-23 pathways. Nat Immunol.

[52] Chikuma S, Suita N, Okazaki IM, Shibayama S, Honjo T (2012). TRIM28 prevents autoinflammatory T cell development *in vivo*. Nat Immunol.

[53] Okamura T, Morita K, Iwasaki Y, Inoue M, Komai T, Fujio K (2015). Role of TGF-β3 in the regulation of immune responses. Clin Exp Rheumatol.

[54] Yamaguchi Y, Tsumura H, Miwa M, Inaba K (2010). Contrasting effects of TGF-β1 and TNF-α on the development of dendritic cells from progenitors in mouse bone marrow. Stem Cells.

[55] Bao M, Wang Y, Liu Y, Shi P, Lu H, Sha W (2016). NFATC3 promotes IRF7 transcriptional activity in plasmacytoid dendritic cells. J Exp Med.

[56] Yang K, Puel A, Zhang S, Eidenschenk C, Ku CL, Casrouge A (2005). Human TLR-7-,-8-, and-9-mediated induction of IFN-α/β and-λ is IRAK-4 dependent and redundant for protective immunity to viruses. Immunity.

[57] Shen J, Wang S, Zhang YJ, Kappil M, Wu HC, Kibriya MG (2012). Genome-wide DNA methylation profiles in hepatocellular carcinoma. Hepatology.

[58] Ye C, Tao R, Cao Q, Zhu D, Wang Y, Wang J (2016). Whole-genome DNA methylation and hydroxymethylation profiling for HBV-related hepatocellular carcinoma. Int J Oncol.

[59] Yu J, Ni M, Xu J, Zhang H, Gao B, Gu J (2002). Methylation profiling of twenty promoter-CpG islands of genes which may contribute to hepatocellular carcinogenesis. BMC Cancer.

[60] Machicado C, Machicado JD, Maco V, Terashima A, Marcos LA (2016). Association of *Fasciola hepatica* infection with liver fibrosis, cirrhosis, and cancer: a systematic review. PLoS Negl Trop Dis.

[61] Almendras-Jaramillo M, Rivera-Medina J, Seijas-Mogrovejo J, Almendras-Jaramillo K (1997). Hepatic fascioliasis in children: uncommon clinical manifestations. Arq Gastroenterol.

[62] Barton BE (2001). IL-6-like cytokines and cancer cachexia: consequences of chronic inflammation. Immunol Res.

[63] Fujiki H, Suganuma M, Okabe S, Kurusu M, Imai K, Nakachi K (2002). Involvement of TNF-α changes in human cancer development, prevention and palliative care. Mech Ageing Dev.

[64] Teng MW, Bowman EP, McElwee JJ, Smyth MJ, Casanova JL, Cooper AM (2015). IL-12 and IL-23 cytokines: from discovery to targeted therapies for immune-mediated inflammatory diseases. Nat Med.

[65] Floros T, Tarhini AA (2015). Anticancer cytokines: Biology and clinical effects of IFN-α2, IL-2, IL-15, IL-21, and IL-12. Semin Oncol.

[66] Hagerling C, Casbon AJ, Werb Z (2015). Balancing the innate immune system in tumor development. Trends Cell Biol.

[67] Porta C, Riboldi E, Sica A (2011). Mechanisms linking pathogens-associated inflammation and cancer. Cancer Lett.

[68] Pal S, Bhattacharjee A, Ali A, Mandal NC, Mandal SC, Pal M (2014). Chronic inflammation and cancer: potential chemoprevention through nuclear factor kappa B and p53 mutual antagonism. J Inflamm.

[69] Belamaric J (1973). Intrahepatic bile duct carcinoma and *C. sinensis* infection in Hong Kong. Cancer.

[70] Sripa B, Brindley PJ, Mulvenna J, Laha T, Smout MJ, Mairiang E (2012). The tumorigenic liver fluke *Opisthorchis viverrini*–multiple pathways to cancer. Trends Parasitol.

[71] Lvova MN, Tangkawattana S, Balthaisong S, Katokhin AV, Mordvinov VA, Sripa B (2012). Comparative histopathology of *Opisthorchis felineus* and *Opisthorchis viverrini* in a hamster model: an implication of high pathogenicity of the European liver fluke. Parasitol Int.

[72] Choi YK, Yoon BI, Won YS, Lee CH, Hyun BH, Kim HC (2003). Cytokine responses in mice infected with *Clonorchis sinensis*. Parasitol Res.

[73] Sripa B, Thinkhamrop B, Mairiang E, Laha T, Kaewkes S, Sithithaworn P (2012). Elevated plasma IL-6 associates with increased risk of advanced fibrosis and cholangiocarcinoma in individuals infected by *Opisthorchis viverrini*. PLoS Negl Trop Dis.

[74] Jittimanee J, Sermswan RW, Puapairoj A, Maleewong W, Wongratanacheewin S (2010). Cytokine expression in hamsters experimentally infected with *Opisthorchis viverrini*. Parasite Immunol.

