# Supplementary figures and images for: DNA methylation and hydroxymethylation profiles reveal possible role of highly methylated TLR signaling on Fasciola gigantica excretory/secretory products (FgESPs) modulation of buffalo dendritic cells
Source: Parasit Vectors. 2019 Jul 23;12:358. doi: 10.1186/s13071-019-3615-4 (PMC6647289; doi:10.1186/s13071-019-3615-4)

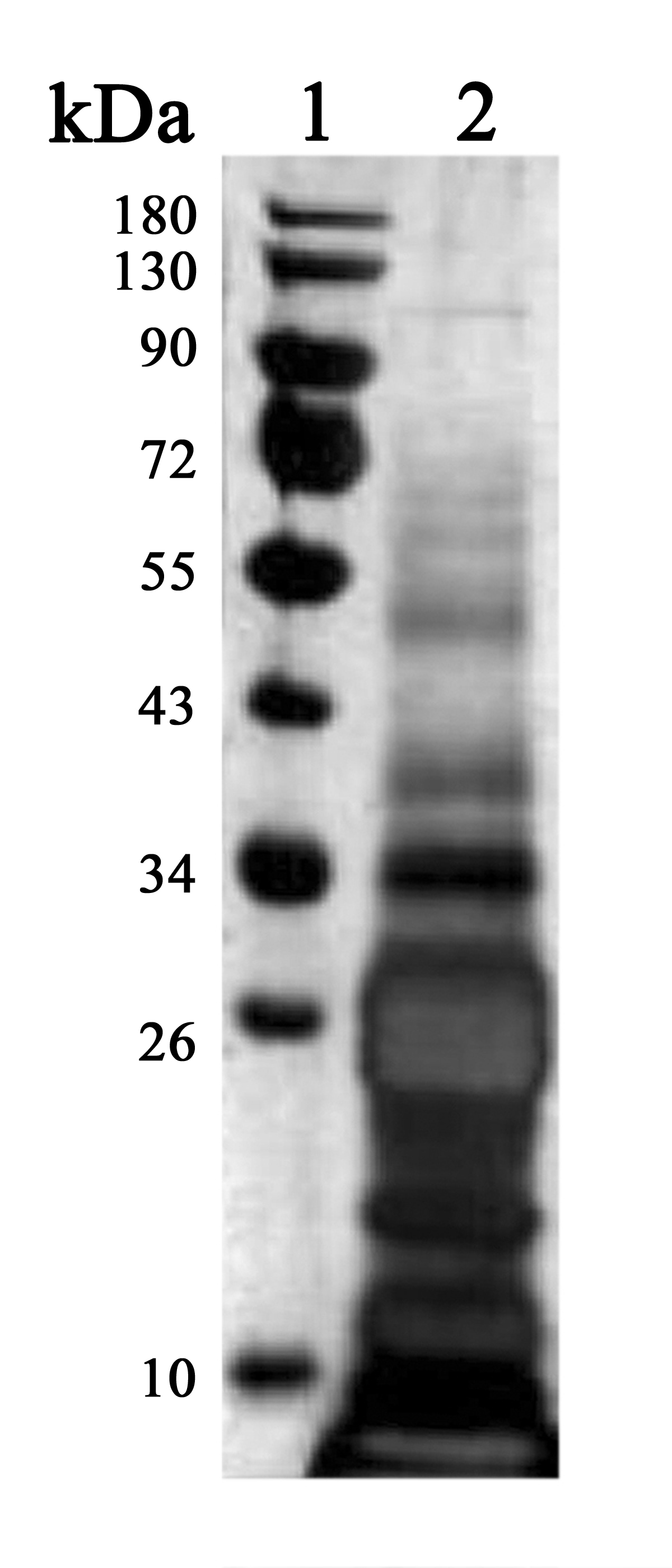

Supplement: Supplementary file 1 — Additional file 1: Figure S1. Molecular weight and protein profile of FgESPs in SDS-PAGE gel. Lane 1: molecular size marker ranging from 10 to 180 kDa (Thermo Fisher Scientific, Wilmington, DE, USA); Lane 2:5 μg of prepared FgESPs. [file 13071_2019_3615_MOESM1_ESM.tif]

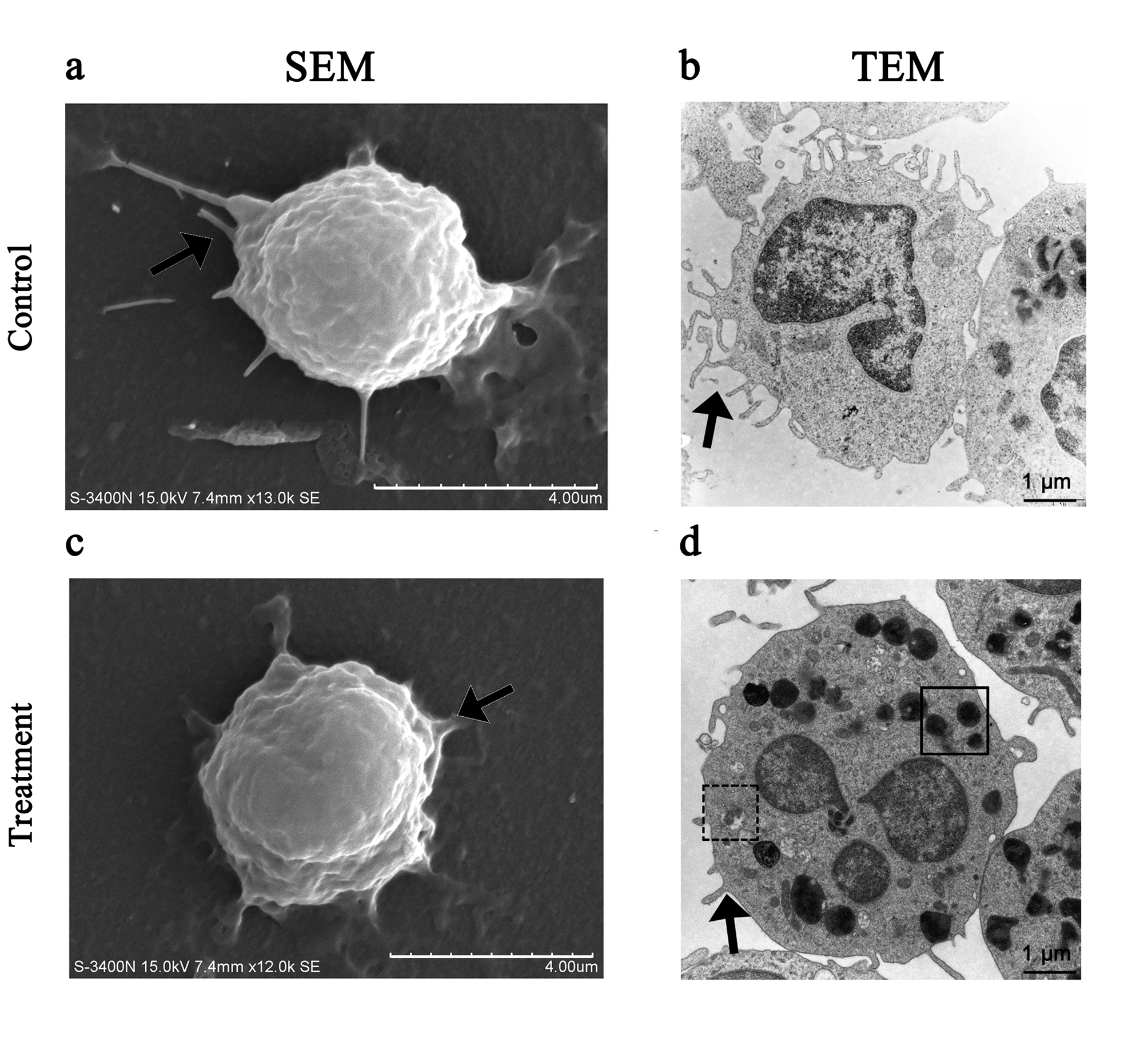

Supplement: Supplementary file 2 — Additional file 2: Figure S2. Ultrastructural changes of buffalo DCs treated with FgESPs. Cell surface and intracellular ultrastructural features of DCs were observed using scanning electron microscopy (SEM) and transmission electron microscopy (TEM), respectively. The results demonstrate the occurrence of several intracellular lysosomes (solid box) and phagosome-like balls (dashed box), as well as fewer synapses (arrows) in DCs treated with FgESPs (c, d) rather than in the control DCs (a, b). Scale-bars: a, c, 4 μm; b, d, 1 μm. [file 13071_2019_3615_MOESM2_ESM.tif]

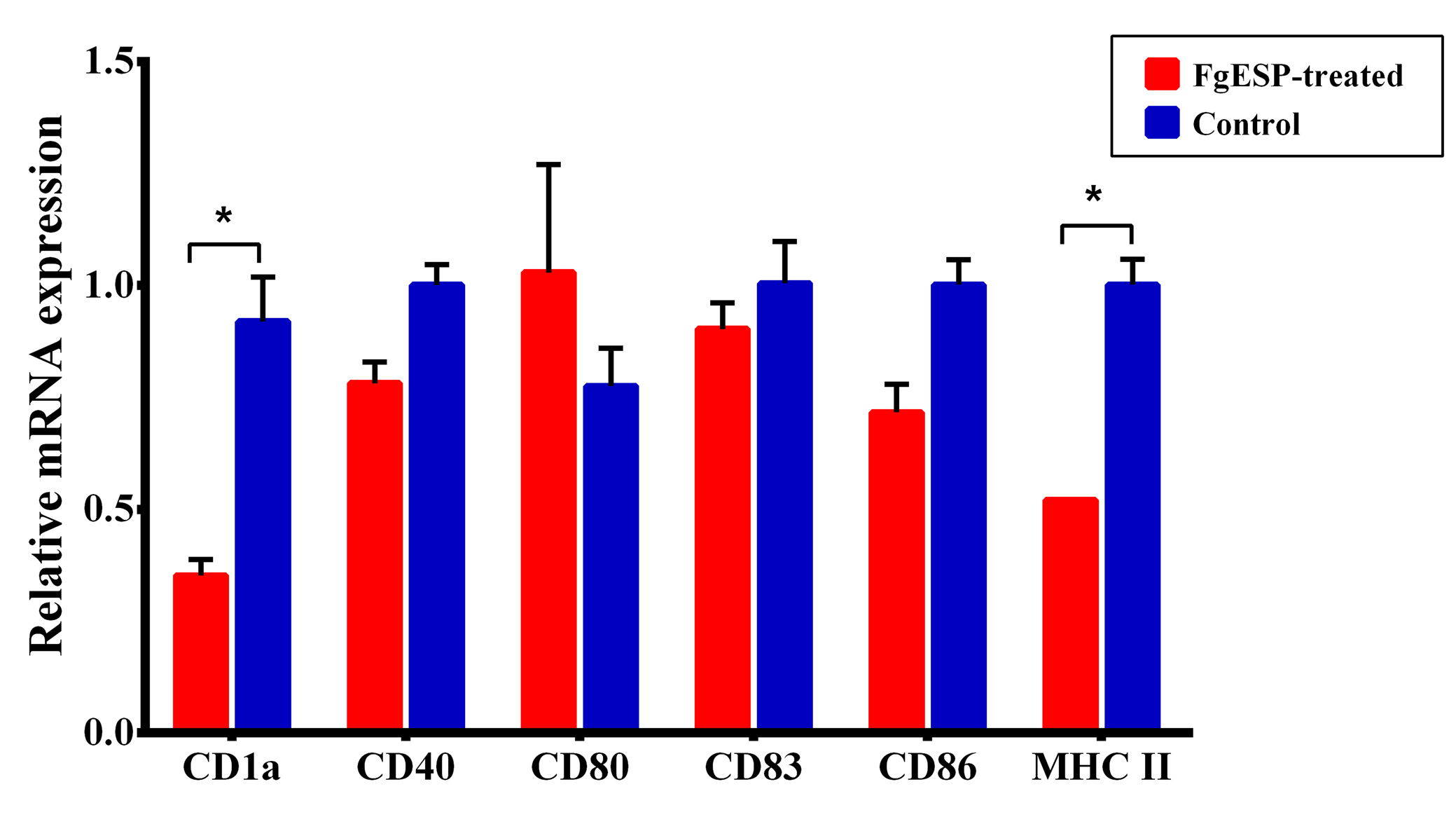

Supplement: Supplementary file 3 — Additional file 3: Figure S3. Changes in mRNA expression of surface markers in buffalo DCs treated with FgESPs. The x-axis indicates the names of genes and the y-axis represents the relative mRNA expression of target genes. Expression is relative to the buffalo GAPDH gene based on the 2−ΔΔCq calculation. Red bars represent the FgESPs-treated groups and blue bars represent the control DCs. Columns show the means, and error bars show SEMs. Significant differences were compared to the control group. *P < 0.05 (Student’s t-test). [file 13071_2019_3615_MOESM3_ESM.tif]
