# Supplementary material for: DNA methylation and hydroxymethylation profiles reveal possible role of highly methylated TLR signaling on Fasciola gigantica excretory/secretory products (FgESPs) modulation of buffalo dendritic cells
Source: Parasit Vectors. 2019 Jul 23;12:358. doi: 10.1186/s13071-019-3615-4 (PMC6647289; doi:10.1186/s13071-019-3615-4)
Supplement: Supplementary file 4 — Additional file 4: Table S1. Pathway analysis of DMR-associated genes. Table S2. Pathway analysis of DhMR-associated genes. [file 13071_2019_3615_MOESM4_ESM.docx]

**Additional file 4: Table S1.** Pathway analysis of DMR-associated genes

| **Pathway name** | **ID** | **Genes** | **Statistic** |
| --- | --- | --- | --- |
| A, Pathway analysis of hypermethylated genes | | | |
| Calcium signaling pathway | 04020 | ADCY2, ADCY7, ADORA2A, ATP2B2, ATP2B4, BDKRB1, CACNA1S, CAMK2B, ERBB3, F2R, GNA14, GNAS, GRIN2D, GRM5, HRH2, HTR5A, LTB4R2, MYLK2, MYLK4, NOS3, ORAI2, P2RX2, PLCG1, PLCG2, PLN, PRKACB, PRKCB, PTAFR, PTGER1, RYR2, SLC25A6, SPHK1, STIM2, TACR2, VDAC1 | O = 35; C = 194; *P* = 3.27e05; F = 0.0100 |
| Platelet activation | 04021 | ADCY2, ADCY7, ARHGAP35, ARHGEF12, F2R, FYN, GNAS, GP1BA, GUCY1A2, MAPK12, MYL12B, MYLK2, MYLK4, NOS3, PIK3R3, PLA2G4B, PLCG2, PRKACB, PRKCZ, PTGS1, RASGRP1, SRC, TLN2, VASP | O = 24; C = 119; *P* = 9.50e05; F = 0.1450 |
| Inflammatory mediator regulation of TRP channels | 04022 | ADCY2, ADCY7, BDKRB1, CAMK2B, F2RL1, GNAS, IL1R1, MAP2K3, MAP2K6, MAPK12, PIK3R3, PLA2G4B, PLA2G6, PLCG1, PLCG2, PRKACB, PRKCB, PRKCD, SRC, TRPV1 | O = 20; C = 105; *P* = 0.0008; F = 0.0784 |
| Cysteine and methionine metabolism | 04023 | AMD1, APIP, BCAT1, CBS, CDO1, DNMT3A, GCLM, LDHB, MPST, MRI1, TST | O = 11; C = 44; *P* = 0.0012; F = 0.0872 |
| Proteasome | 04024 | PSMA8, PSMB2, PSMB3, PSMB6, PSMC2, PSMC4, PSMC5, PSMD1, PSMD6, PSMD8, PSME2 | O = 11; C = 45; *P* = 0.0015; F = 0.0872 |
| Ras signaling pathway | 04025 | ANGPT4, BCL2L1, EFNA4, FASLG, FGF14, FGF6, FGF7, FGFR4, GNGT1, GNGT2, GRB2, IKBKB, KSR1, LAT, PDGFD, PIK3R3, PLA1A, PLA2G2C, PLA2G4B, PLA2G5, PLA2G6, PLCG1, PLCG2, PRKACB, PRKCB, RAB5B, RALB, RAPGEF5, RASGRP1, SHC3, SHOC2, SOS1, SYNGAP1, TEK, TIAM1, ZAP70 | O = 36; C = 246; *P* = 0.0017; F = 0.0872 |
| Regulation of actin cytoskeleton | 04026 | ACTN1, ARHGAP35, ARHGEF12, ARHGEF7, ARPC1B, BDKRB1, DIAPH1, F2, F2R, FGF14, FGF6, FGF7, FGFR4, GNA12, IQGAP3, ITGA8, MYL10, MYL12B, MYL2, MYLK2, MYLK4, PDGFD, PIK3R3, PPP1R12B, PPP1R12B, PXN, SCIN, SOS1, SRC, SSH3, TIAM1, VAV2 | O = 37; C = 215; *P* = 1.21e05; F = 0.0037 |
| Oxytocin signaling pathway | 04027 | ADCY2, ADCY7, CACNA1S, CACNA2D4, CACNB3, CACNG5, CAMK2B, GNAS, GUCY1A2, KCNJ14, MEF2C, MYLK2, MYLK4, NOS3, NPR2, PIK3R3, PLA2G4B, PPP1R12B, PPP1R12B, PRKAA1, PRKACB, PRKAG3, PRKCB, RYR2, SRC | O = 25; C = 160; *P* = 0.0035; F = 0.1294 |
| Proteoglycans in cancer | 04028 | ARHGEF12, CAMK2B, CASP3, ERBB3, FASLG, FZD9, GRB2, HPSE, IL12B, MAPK12, MTOR, PIK3R3, PLCG1, PLCG2, PPP1R12B, PPP1R12B, PRKACB, PRKCB, PXN, SOS1, SRC, TIAM1, TLR2, TLR4, TWIST2, VTN, WNT1, WNT2B, WNT5B, WNT7A | O = 30; C = 205; *P* = 0.0040; F = 0.1294 |
| Neuroactive ligand-receptor interaction | 04029 | ADORA1, ADORA2A, BDKRB1, CHRNA10, CHRNG, CRHR1, F2, F2R, F2RL1, FSHR, GABRR2, GABRR3, GIPR, GLP1R, GLRA3, GPR50, GRIA3, GRID2, GRIK5, GRIN2D, GRM5, GRM6, HRH2, HTR5A, KISS1R, LHB, LPAR2, LTB4R2, MLNR, MTNR1B, P2RX2, P2RY11, P2RY13, P2RY8, PRLHR, PTAFR, PTGER1, RXFP3, TACR2, TRPV1, VIPR2 | O = 41; C = 305; *P* = 0.0042; F = 0.1294 |
| Gastric acid secretion | 04030 | ADCY2, ADCY7, ATP4A, CAMK2B, GNAS, HRH2, KCNJ1, KCNJ16, KCNK10, KCNK2, MYLK2, MYLK4, PRKACB, PRKCB | O = 14; C = 74; *P* = 0.0049; F = 0.1325 |
| Chemokine signaling pathway | 04031 | ADCY2, ADCY7, ARRB1, CCL19, CCL26, CCR9, CXCL14, CXCL16, CXCR3, FOXO3, GNGT1, GNGT2, GRB2, GRK4, GRK6, IKBKB, PIK3R3, PREX1, PRKACB, PRKCB, PRKCD, PRKCZ, PXN, SHC3, SOS1, SRC, TIAM1, VAV2 | O = 28; C = 191; *P* = 0.0052; F = 0.1325 |
| Fat digestion and absorption | 04032 | ABCG5, ABCG8, AGPAT1, APOA1, APOA4, CEL, NPC1L1, PLA2G2C, PLA2G5, PNLIP, PNLIPRP2 | O = 11; C = 53; *P* = 0.0058; F = 0.1372 |
| GnRH signaling pathway | 04033 | ADCY2, ADCY7, CACNA1S, CAMK2B, GNAS, GRB2, LHB, MAP2K3, MAP2K6, MAPK12, PLA2G4B, PRKACB, PRKCB, PRKCD, SOS1, SRC | O = 16; C = 93; *P* = 0.0071; F = 0.1520 |
| Focal adhesion | 04034 | ACTN1, ARHGAP35, CCND3, COL6A6, COL9A2, DIAPH1, FYN, GRB2, ITGA8, MYL10, MYL12B, MYL2, MYLK2, MYLK4, PARVB, PARVG, PDGFD, PIK3R3, PPP1R12B, PPP1R12B, PRKCB, PXN, SHC3, SOS1, SRC, TLN2, VASP, VAV2, VTN | O = 29; C = 205; *P* = 0.0075; F = 0.1520 |
| Vascular smooth muscle contraction | 04035 | ADCY2, ADCY7, ADORA2A, ARHGEF12, CACNA1S, GNA12, GNAS, GUCY1A2, MYLK2, MYLK4, NPR2, PLA2G2C, PLA2G4B, PLA2G5, PLA2G6, PPP1R12B, PPP1R12B, PRKACB, PRKCB, PRKCD | O = 20; C = 128; *P* = 0.0085; F = 0.1615 |
| NF-kappa B signaling pathway | 04064 | BCL2L1, CCL19, CSNK2B, ERC1, IKBKB, IL1R1, IRAK1, LAT, PIAS4, PLCG1, PLCG2, PRKCB, TICAM2, TGF4, ZAP70 | O = 15; C = 101; *P* = 0.0316; F = 0.3007 |
| B, Pathway analysis of hypomethylated genes | | | |
| Regulation of actin cytoskeleton | 04810 | ACTN1, ARAF, ARHGAP35, ARHGEF7, ARPC1B, ARPC5L, BCAR1, CRK, F2, F2R, FGF1, FGF21, FGF6, HRAS, INS, INSRR, ITGA10, ITGA7, ITGB2, ITGB7, KRAS, MAPK1, MOS, MYL10, MYLK, MYLK2, MYLK4, PAK2, PDGFA, PIK3CA, PIK3R3, PPP1CB, PXN, RDX, TIAM1, VAV2, WASF1 | O = 37; C = 215; *P* = 1.21e05; F = 0.0037 |
| TGF-beta signaling pathway | 04350 | ACVR2B, BAMBI, BMP4, BMPR1B, BMPR2, DCN, ID2, INHBE, MAPK1, PITX2, PPP2R1B, RPS6KB2, SMAD9, TFDP1, TGFB3, THBS1 | O = 16; C = 83; *P* = 0.0011; F = 0.0769 |
| Focal adhesion | 04510 | ACTN1, ARHGAP35, BCAR1, COL6A2, COL9A2, CRK, HRAS, ITGA10, ITGA7, ITGB7, KDR, LAMB2, MAPK1, MYL10, MYLK, MYLK2, MYLK4, PAK2, PDGFA, PDPK1, PIK3CA, PIK3R3, PPP1CB, PXN, RAPGEF1, THBS1, THBS3, TLN2, TNXB, VAV2 | O = 30; C = 205; *P* = 0.0014; F = 0.0769 |
| Pathways in cancer | 05200 | ADCY9, AGTR1, ARAF, ARHGEF11, BCR, BMP4, CASP8, CEBPA, CHUK, CRK, CTBP2, DVL1, EGLN2, F2R, F2RL3, FADD, FGF1, FGF21, FGF6, FZD6, GNAI2, GNAS, GNG4, HDAC2, HRAS, HSP90AA1, KRAS, LAMB2, MAPK1, MECOM, MITF, MMP9, MSH3, PDGFA, PIK3CA, PIK3R3, PLCG1, PTCH1, RARB, RASGRP4, RUNX1T1, SPI1, TCF7L2, TGFB3, TP53, TRAF2, TRAF3, WNT4, WNT8A, WNT8B, WNT9A | O = 51; C = 409; *P* = 0.0017; F = 0.0769 |
| Glycosphingolipid biosynthesis | 00603 | A3GALT2, B3GALNT1, B3GALT5, FUT1, HEXB, NAGA | O = 6; C = 17; *P* = 0.0017; F = 0.0769 |
| MAPK signaling pathway | 04010 | ARRB2, CACNA1B, CACNA1E, CACNB2, CACNG4, CHUK, CRK, DUSP6, FGF1, FGF21, FGF6, HRAS, HSPA1L, IL1R1, KRAS, MAP2K4, MAP3K6, MAP3K8, MAP4K4, MAPK1, MECOM, MOS, NFATC3, NLK, NR4A1, PAK2, PDGFA, PPP3CB, RASGRF2, RASGRP4, RPS6KA2, RPS6KA5, TGFB3, TP53, TRAF2 | O = 35; C = 256; *P* = 0.0019; F = 0.0769 |
| Prostate cancer | 05215 | ARAF, CHUK, CREB3L1, HRAS, HSP90AA1, INS, INSRR, KRAS, MAPK1, PDGFA, PDPK1, PIK3CA, PIK3R3, SRD5A2, TCF7L2, TP53 | O = 16; C = 89; *P* = 0.0023; F = 0.0769 |
| Acute myeloid leukemia | 05221 | ARAF, CEBPA, CHUK, HRAS, KRAS, MAPK1, PIK3CA, PIK3R3, RPS6KB2, RUNX1T1, SPI1, TCF7L2 | O = 12; C = 58; *P* = 0.0023; F = 0.0769 |
| Wnt signaling pathway | 04310 | BAMBI, BTRC, CAMK2G, CTBP2, CTNNBIP1, DVL1, FZD6, NFATC3, NKD2, NLK, PPP3CB, PRICKLE2, RUVBL1, SIAH1, TCF7L2, TP53, VANGL1, VANGL2, WIF1, WNT4, WNT8A, WNT8B, WNT9A | O = 23; C = 149; *P* = 0.0024; F = 0.0769 |
| Chronic myeloid leukemia | 05220 | ARAF, BCR, CHUK, CRK, CTBP2, HDAC2, HRAS, KRAS, MAPK1, MECOM, PIK3CA, PIK3R3, TGFB3, TP53 | O = 14; C = 74; *P* = 0.0026; F = 0.0769 |
| Proteoglycans in cancer | 05205 | ARAF, CAMK2G, DCN, ERBB4, FZD6, HPSE, HRAS, KDR, KRAS, MAPK1, MMP9, PDPK1, PIK3CA, PIK3R3, PLAUR, PLCG1, PPP1CB, PTCH1, PXN, RDX, RPS6KB2, THBS1, TIAM1, TP53, WNT4, WNT8A, WNT8B, WNT9A | O = 29; C = 205; *P* = 0.0028; F = 0.0769 |
| Neurotrophin signaling pathway | 04722 | ARHGDIA, CAMK2G, CRK, FOXO3, HRAS, IRAK4, KRAS, MAPK1, NFKBIB, PDPK1, PIK3CA, PIK3R3, PLCG1, PRDM4, RAPGEF1, RPS6KA2, RPS6KA5, SH2B2, TP53, TP73 | O = 20; C = 126; *P* = 0.0032; F = 0.0776 |
| TNF signaling pathway | 04668 | CASP8, CHUK, CREB3L1, CSF2, DAB2IP, DNM1L, FADD, IL18R1, MAP2K4, MAP3K8, MAPK1, MMP9, PGAM5, PIK3CA, PIK3R3, RPS6KA5, TRAF2, TRAF3 | O = 18; C = 109; *P* = 0.0033; F = 0.0776 |
| mTOR signaling pathway | 04150 | ATP6V1G3, CHUK, CLIP1, DDIT4, DVL1, FZD6, HRAS, INS, KRAS, MAPK1, MLST8, PDPK1, PIK3CA, PIK3R3, RPS6KA2, RPS6KB2, RRAGA, SEC13, TBC1D7, WNT4, WNT8A, WNT8B, WNT9A | O = 23; C = 157; *P* = 0.0047; F = 0.1030 |
| Signaling pathways regulating pluripotency of stem cells | 04550 | ACVR2B, BMP4, BMPR1B, BMPR2, DVL1, FZD6, HRAS, ID2, INHBE, KRAS, MAPK1, MYF5, PIK3CA, PIK3R3, REST, SMAD9, TBX3, WNT4, WNT8A, WNT8B, WNT9A | O = 21; C = 141; *P* = 0.0056; F = 0.1098 |
| Axon guidance | 04360 | ABLIM2, BMPR1B, BMPR2, CAMK2G, EPHA8, GNAI2, HRAS, KRAS, LRRC4, MAPK1, NFATC3, PAK2, PARD6B, PIK3CA, PIK3R3, PLCG1, PLXNB1, PLXNC1, PPP3CB, PTCH1, RGS3, SEMA3D, SEMA4A, SRGAP3, WNT4 | O = 25; C = 178; *P* = 0.0058; F = 0.1098 |
| Complement and coagulation cascades | 04610 | C8G, F2, F2R, F2RL3, F5, F7, ITGB2, MASP1, MASP2, PLAUR, SERPINA5, SERPINC1, SERPIND1, SERPINE1, SERPINF2 | O = 15; C = 90; *P* = 0.0065; F = 0.1098 |
| Leukocyte transendothelial migration | 04670 | ACTN1, ARHGAP35, BCAR1, CDH5, CLDN10, CLDN14, CLDN19, CTNND1, GNAI2, ITGB2, MMP9, MYL10, PIK3CA, PIK3R3, PLCG1, PXN, THY1, VAV2 | O = 18; C = 116; *P* = 0.0065; F = 0.1098 |
| Non-small cell lung cancer | 05223 | ARAF, FOXO3, HRAS, KRAS, MAPK1, PDPK1, PIK3CA, PIK3R3, PLCG1, RARB, TP53 | O = 11; C = 58; *P* = 0.0070; F = 0.1126 |
| Hippo signaling pathway | 04390 | BMP4, BMPR1B, BMPR2, BTRC, DVL1, FGF1, FZD6, ID2, ITGB2, MOB1B, PARD6B, PPP1CB, PPP2R1B, SERPINE1, TCF7L2, TGFB3, TP53BP2, TP73, WNT4, WNT8A, WNT8B, WNT9A | O = 22; C = 154; *P* = 0.0076; F = 0.1155 |
| Longevity regulating pathway | 04213 | ADCY9, FOXO3, HDAC2, HRAS, HSPA1L, INS, KRAS, PIK3CA, PIK3R3, PRKAG2, PRKAG3, RPS6KB2 | O = 12; C = 67; *P* = 0.0080; F = 0.1155 |
| T cell receptor signaling pathway | 04660 | CHUK, CSF2, HRAS, KRAS, LCK, MAP3K8, MAPK1, NFATC3, NFKBIB, PAK2, PDPK1, PIK3CA, PIK3R3, PLCG1, PPP3CB, PRKCQ, VAV2 | O = 17; C = 110; *P* = 0.0083; F = 0.1157 |
| Melanogenesis | 04916 | ADCY9, CAMK2G, CREB3L1, DVL1, FZD6, GNAI2, GNAS, HRAS, KRAS, MAPK1, MITF, TCF7L2, WNT4, WNT8A, WNT8B, WNT9A | O = 16; C = 103; *P* = 0.0098; F = 0.1303 |

*Abbreviations*: O, the number of the DE genes' entities directly associated with the listed category; C, the count of the chosen background population genes' entities associated with the listed category; *P*, the enrichment *P*-value of the category used Fisher's exact test; E, the Enrichment Score value, it equals "-log10 (*P*-value)"; F, the false discovery rate.

**Additional file 4: Table S2.** Pathway analysis of DhMR-associated genes

| **Pathway name** | **ID** | **Genes** | **Statistic** |
| --- | --- | --- | --- |
| A, Pathway analysis of upregulated DhMR-associated genes | | | |
| Type II diabetes mellitus | 04930 | ABCC8, CACNA1A, TNF | O = 3; C = 46; *P* = 0.0009; F = 0.2525 |
| Legionellosis | 05134 | HSPA1L, SAR1B, TNF | O = 3; C = 56; *P* = 0.0016; F = 0.2525 |
| Bacterial invasion of epithelial cells | 05100 | ARHGAP10, PTK2, SEPT8 | O = 3; C = 77; *P* = 0.0040; F = 0.4184 |
| MAPK signaling pathway | 04010 | CACNA1A, HSPA1L, NFATC3, TAOK1, TNF | O = 5; C = 294; *P* = 0.0071; F = 0.5594 |
| Human papillomavirus infection | 05165 | MDM2, MPP5, PTK2, TNF, UBE3A | O = 5; C = 338; *P* = 0.0125; F = 0.6664 |
| Nicotine addiction | 05033 | CACNA1A, GRIN2D | O = 2; C = 42; *P* = 0.0132; F = 0.6664 |
| Bladder cancer | 05219 | DAPK3, MDM2 | O = 2; C = 46; *P* = 0.0157; F = 0.6664 |
| Cocaine addiction | 05030 | GRIN2D, PDYN | O = 2; C = 49; *P* = 0.0177; F = 0.6664 |
| Amyotrophic lateral sclerosis | 05014 | GRIN2D, TNF | O = 2; C = 51; *P* = 0.0191; F = 0.6664 |
| Ubiquitin mediated proteolysis | 04120 | MDM2, UBE2B, UBE3A | O = 3; C = 142; *P* = 0.0211; F = 0.6664 |
| Hepatitis B | 05161 | EGR3, NFATC3, TNF | O = 3; C = 155; *P* = 0.0265; F = 0.7338 |
| cGMP-PKG signaling pathway | 04022 | NFATC3, NPR1, SLC25A6 | O = 3; C = 165; *P* = 0.0311; F = 0.7338 |
| Cellular senescence | 04218 | MDM2, NFATC3, SLC25A6 | O = 3; C = 166; *P* = 0.0316; F = 0.7338 |
| Amphetamine addiction | 05031 | GRIN2D, PDYN | O = 2; C = 68; *P* = 0.0326; F = 0.7338 |
| Fc epsilon RI signaling pathway | 04664 | INPP5D, TNF | O = 2; C = 73; *P* = 0.0371; F = 0.7338 |
| Alzheimer's disease | 05010 | APBB1, GRIN2D, TNF | O = 3; C = 177; *P* = 0.0372; F = 0.7338 |
| B cell receptor signaling pathway | 04662 | INPP5D, NFATC3 | O = 2; C = 79; *P* = 0.0429; F = 0.7585 |
| Influenza A | 05164 | HSPA1L, SLC25A6, TNF | O = 3; C = 188; *P* = 0.0432; F = 0.7585 |
| Calcium signaling pathway | 04020 | CACNA1A, GRIN2D, SLC25A6 | O = 3; C = 194; *P* = 0.0467; F = 0.7768 |
| Antigen processing and presentation | 04612 | HSPA1L, TNF | O = 2; C = 86; *P* = 0.0499; F = 0.7857 |
| B, Pathway analysis of downregulated DhMR-associated genes | | | |
| RNA transport | 03013 | EIF1B, EIF2B1, EIF4G3, RBM8A | O = 4; C = 171; *P* = 0.0054; F = 0.8142 |
| RNA polymerase | 03020 | POLR1A, POLR3GL | O = 2; C = 30; *P* = 0.0069; F = 0.8142 |
| Estrogen signaling pathway | 04915 | CREB3L1, HSPA1L, MAP2K2 | O = 3; C = 99; *P* = 0.0080; F = 0.8142 |
| Aminoacyl-tRNA biosynthesis | 00970 | SEPSECS, VARS | O = 2; C = 44; *P* = 0.0144; F = 0.8142 |
| Neurotrophin signaling pathway | 04722 | IRAK4, MAP2K2, RAP1B | O = 3; C = 124; *P* = 0.0147; F = 0.8142 |
| Cocaine addiction | 05030 | CREB3L1, PDYN | O = 2; C = 49; *P* = 0.0177; F = 0.8142 |
| Antifolate resistance | 01523 | ABCG2, SLC46A1 | O = 2; C = 51; *P* = 0.0191; F = 0.8142 |
| VEGF signaling pathway | 04370 | MAP2K2, SPHK2 | O = 2; C = 60; *P* = 0.0259; F = 0.8142 |
| Protein processing in endoplasmic reticulum | 04141 | CALR, HSPA1L, SEC24A | O = 3; C = 166; *P* = 0.0316; F = 0.8142 |
| Amphetamine addiction | 05031 | CREB3L1, PDYN | O = 2; C = 68; *P* = 0.0326; F = 0.8142 |
| Acute myeloid leukemia | 05221 | MAP2K2, RUNX1T1 | O = 2; C = 68; *P* = 0.0326; F = 0.8142 |
| MAPK signaling pathway | 04010 | HSPA1L, IRAK4, MAP2K2, RAP1B | O = 4; C = 294; *P* = 0.0333; F = 0.8142 |
| Long-term potentiation | 04720 | MAP2K2, RAP1B | O = 2; C = 69; *P* = 0.0335; F = 0.8142 |
| Renal cell carcinoma | 05211 | MAP2K2, RAP1B | O = 2; C = 73; *P* = 0.0371; F = 0.8382 |
| Influenza A | 05164 | HSPA1L, IRAK4, MAP2K2 | O = 3; C = 188; *P* = 0.0432; F = 0.8857 |
| Aldosterone synthesis and secretion | 04925 | CREB3L1, DAGLA | O = 2; C = 81; *P* = 0.0448; F = 0.8857 |
| Antigen processing and presentation | 04612 | CALR, HSPA1L | O = 2; C = 86; *P* = 0.0499; F = 0.9056 |

*Abbreviations*: O, the number of the DE genes' entities directly associated with the listed category; C, the count of the chosen background population genes' entities associated with the listed category; *P*, the enrichment *P*-value of the category used Fisher's exact test; E, the Enrichment Score value, it equals "-log10 (*P*-value)"; F, the false discovery rate.
